# Synthesis of Galactosyl‐Queuosine and Distribution of Hypermodified Q‐Nucleosides in Mouse Tissues

**DOI:** 10.1002/anie.202002295

**Published:** 2020-04-21

**Authors:** Peter Thumbs, Timm T. Ensfelder, Markus Hillmeier, Mirko Wagner, Matthias Heiss, Constanze Scheel, Alexander Schön, Markus Müller, Stylianos Michalakis, Stefanie Kellner, Thomas Carell

**Affiliations:** ^1^ Department of Chemistry Ludwig-Maximilians-Universität München Butenandtstr. 5–13 81377 Munich Germany; ^2^ Department of Pharmacy Ludwig-Maximilians-Universität München Butenandtstr. 5–13 81377 Munich Germany; ^3^ Department of Ophthalmology Ludwig-Maximilians-Universität München Mathildenstr. 8 80336 Munich Germany

**Keywords:** galactosylation, mannosylation, mannosyl-queuosine, queuosine, RNA modifications

## Abstract

Queuosine (Q) is a hypermodified RNA nucleoside that is found in tRNA^His^, tRNA^Asn^, tRNA^Tyr^, and tRNA^Asp^. It is located at the wobble position of the tRNA anticodon loop, where it can interact with U as well as C bases located at the respective position of the corresponding mRNA codons. In tRNA^Tyr^ and tRNA^Asp^ of higher eukaryotes, including humans, the Q base is for yet unknown reasons further modified by the addition of a galactose and a mannose sugar, respectively. The reason for this additional modification, and how the sugar modification is orchestrated with Q formation and insertion, is unknown. Here, we report a total synthesis of the hypermodified nucleoside galactosyl‐queuosine (galQ). The availability of the compound enabled us to study the absolute levels of the Q‐family nucleosides in six different organs of newborn and adult mice, and also in human cytosolic tRNA. Our synthesis now paves the way to a more detailed analysis of the biological function of the Q‐nucleoside family.

## Graphical abstract

**The first total synthesis** of the hypermodified RNA nucleoside galactosyl‐queuosine (galQ) was achieved. Moreover, the distribution of this nucleoside as well as mannosyl‐queuosine and queuosine was determined in tissues of newborn and adult mice, and in human cytosolic tRNAs.

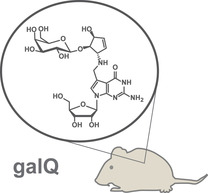

In all three domains of life, RNA contains next to the canonical bases (A, C, G, and U) a large variety of modified nucleosides.^1^ Most of these are chemically simple derivatives of the canonical nucleosides. They often carry methylations at various positions of the heterocycle or the sugar, but others are heavily modified, involving multistep biosynthesis pathways. Queuosine **1** (Q) is one of the most complex of these so‐called hypermodified nucleosides. (Figure 1). It is found in a large number of different species and also present in the cytosolic and mitochondrial tRNA^Tyr^, tRNA^Asp^, tRNA^His^, and tRNA^Asn^ of humans.^2^, ^3^, ^4^, ^5^, ^6^, ^7^, ^8^ Interestingly, in the human cytosolic tRNA^Tyr^ and tRNA^Asp^, Q is further modified with galactose (galQ) and mannose (manQ), respectively.^9^, ^10^ In these tRNAs, the sugar is proposed to be attached to the homoallylic hydroxyl group of the cyclopentene ring system that is linked to the 7‐deazaheterocycle.^11^ While the chemical synthesis of Q has been achieved,^12^, ^13^, ^14^ no reports exist about the preparation of its sugar‐modified derivatives galQ **2** and manQ **3**, which has hampered investigations of their biological role. Accordingly, the exact function of galQ and manQ as part of the human cytosolic tRNA^Tyr^ and tRNA^Asp^ is unknown. In addition, we do not know to which extent the corresponding tRNAs are modified with different Q‐family nucleosides, and how the G/Q‐exchange process and the sugar derivatization is orchestrated. Furthermore, quantitative data about Q‐modification levels in different organs is also lacking.

**Figure 1:**
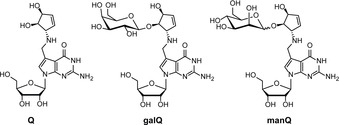
Depiction of the hypermodified RNA nucleoside queuosine (**1**, Q) and of the galactosylated and mannosylated Q derivatives galQ (**2**) and manQ (**3**) present in human cytosolic tRNA^Tyr^ and tRNA^Asp^, respectively.

To address these questions, we performed the first total synthesis of galactosyl‐queuosine **2**. This allowed us to confirm its proposed structure and to report the absolute levels of all Q‐family members in different tissues of newborn and adult mice. Finally, we were able to measure to which extent human cytosolic tRNAs are modified with the three Q‐family nucleosides.

Galactosyl‐Q **2** was constructed from three appropriately protected parts (Figure 2): The 7‐formyl‐7‐deazaguanosine **6** was prepared, as reported by us, with Bz‐protected hydroxyl groups at the ribose, and a pivaloylate protection group at the 2‐amino residue.^14^ The galactose sugar was introduced as a TBS‐ and 2‐chloroisobutyryl‐protected trichloroacetimidate **4**, and the cyclopentene unit **5** was used with Fmoc‐protected allyl amine and a TBS‐protected allylic alcohol. We choose the 2‐chlorobutyryl protecting group for the sugar‐donor **4** because of its bulkiness in order to avoid unwanted orthoester formation as the main product of the glycosylation reaction, a strategy reported by Szpilman et al.^15^

**Figure 2:**
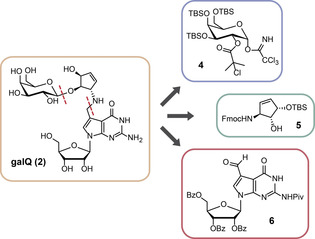
Retrosynthetic analysis for galQ **2**, showing the three key precursors **4**, **5**, and **6**.

The galactosyl‐donor **4** itself was prepared from d‐galactal **7**, which was first TBS‐protected (Scheme 1 A).^16^ *cis*‐Dihydroxylation of the double bond from the sterically less shielded side furnished compound **8**.^17^ This step was followed by protection of the two newly introduced hydroxyl groups with 2‐chloroisobutyric acid to give the galactose‐donor precursor **9**. Deprotection of the anomeric hydroxyl group with hydrazine provided the galactose precursor with a free anomeric hydroxyl group which was subsequently converted into the trichloroacetimidate donor **4** using a standard procedure.

**Scheme 1:**
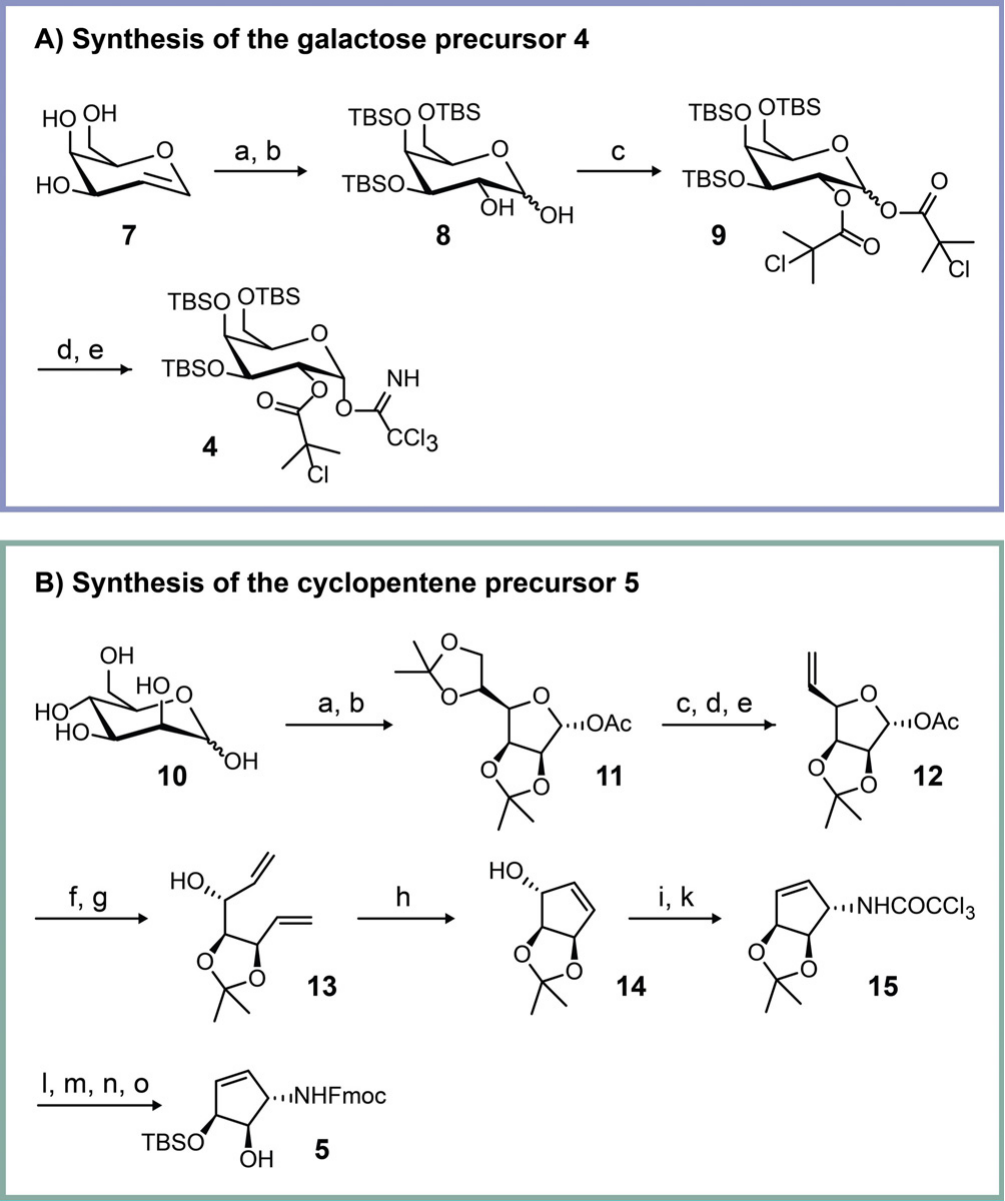
Synthesis of the key precursors **4** and **5**. A) Synthesis of the galactose precursor **4**: a) TBSCl, imidazole, DMF, 55 °C, 2 d; b) K_2_OsO_4_⋅2 H_2_O, NMO, THF, *t*‐BuOH, H_2_O, rt, 4 h; c) 2‐chloroisobutyric acid, DIC, DMAP, 0 °C, 30 min→rt, 2 h; d) N_2_H_4_⋅AcOH, DMF, −40 °C to rt, 3 h; e) Cl_3_CCN, Cs_2_CO_3_, DCM, rt, 4 h. B) Synthesis of the cyclopentene precursor **5**: a) 2,2‐dimethoxypropane, acetone, *p*‐TsOH, rt, 1 h; b) Ac_2_O, pyridine, 0 °C→rt, 18 h; c) aq. AcOH (66 %), 55 °C, 4 h; d) triethylorthoformate, 100 °C, 30 min; e) Ac_2_O, 130 °C, 5 h; f) *t*‐BuOK, MeOH, 20 min; g) NaH, DMSO, Ph_3_PMeBr, THF, rt→68 °C, 2 h; h) Grubbs(I) catalyst, DCM, rt, 26 h; i) Cl_3_CCN, DBU, DCM, rt, 20 min; k) *o*‐xylene, 150 °C, 5 h; l) NaOH, MeOH, rt, overnight; m) Fmoc‐OSu, NaHCO_3_, H_2_O, 1,4‐dioxane; n) AcOH, H_2_O, EtOAc, 50 °C, 24 h; o) TBSOTf, DMF, −55 °C, 15 min.

Scheme 1 B shows the synthesis of the protected 5(*S*)‐amino‐3(*S*),4(*R*)‐dihydroxycyclopent‐1‐ene **5**. The starting point was mannose **10**,^18^ which was converted as reported into the double‐acetonide‐protected mannofuranoside **11** with an acetyl‐protected anomeric center in two steps. Selective cleavage of the acetonide protecting group at the primary hydroxyl group, followed by an orthoester‐based elimination, allowed introduction of a terminal double bond (**12**). Anomeric deprotection, followed by a Wittig reaction, provided the precursor **13** for the ring‐closing metathesis reaction. The free hydroxyl group in **14** was then the starting point for an Overman rearrangement, providing the amine protected as the trichloroacetamide‐protected amine **15**.^19^ Cleavage of this protecting group with NaOH was followed by Fmoc protection of the free amine using a standard procedure. We finally opened the acetal and protected the allylic hydroxyl group selectively with TBS‐OTf in DMF at −55 °C. In this reaction, the temperature is particularly important. When the reaction was performed at higher temperatures and with prolonged reaction times, we noted selective protection of the homoallylic position.

The assembly of the galQ nucleoside **2** from the precursors **4**–**6** is shown in Scheme 2. We first galactosylated the cyclopentene derivative **5**. This sterically demanding step was successfully achieved by activation of the trichloroacetimidate with 2‐chloro‐6‐methylpyridinium triflate in dichloromethane at room temperature.^15^, ^20^ We achieved selective formation of the β‐configured galactoside due to the neighboring‐group effect. Subsequent cleavage of the Fmoc protection group gave product **16**, which was followed by a two‐step reductive amination. First, the imine was formed in benzene, subsequently followed by reduction of the imine with NaBH_4_ in methanol to afford protected galQ **17**. In a two‐step deprotection protocol, we first removed the TBS groups with HF⋅NEt_3_, followed by cleavage of ester‐type protecting groups under Zemplén conditions. For the cleavage of the pivaloyl amide protecting group, we needed to use 0.5 m NaOMe in methanol. This strategy provided the target compound **2** with an overall yield of 0.5 % in 20 linear steps from the mannose starting molecule for the cyclopentene unit. The synthesis provided a sufficient amount of material for all further investigations.

**Scheme 2:**
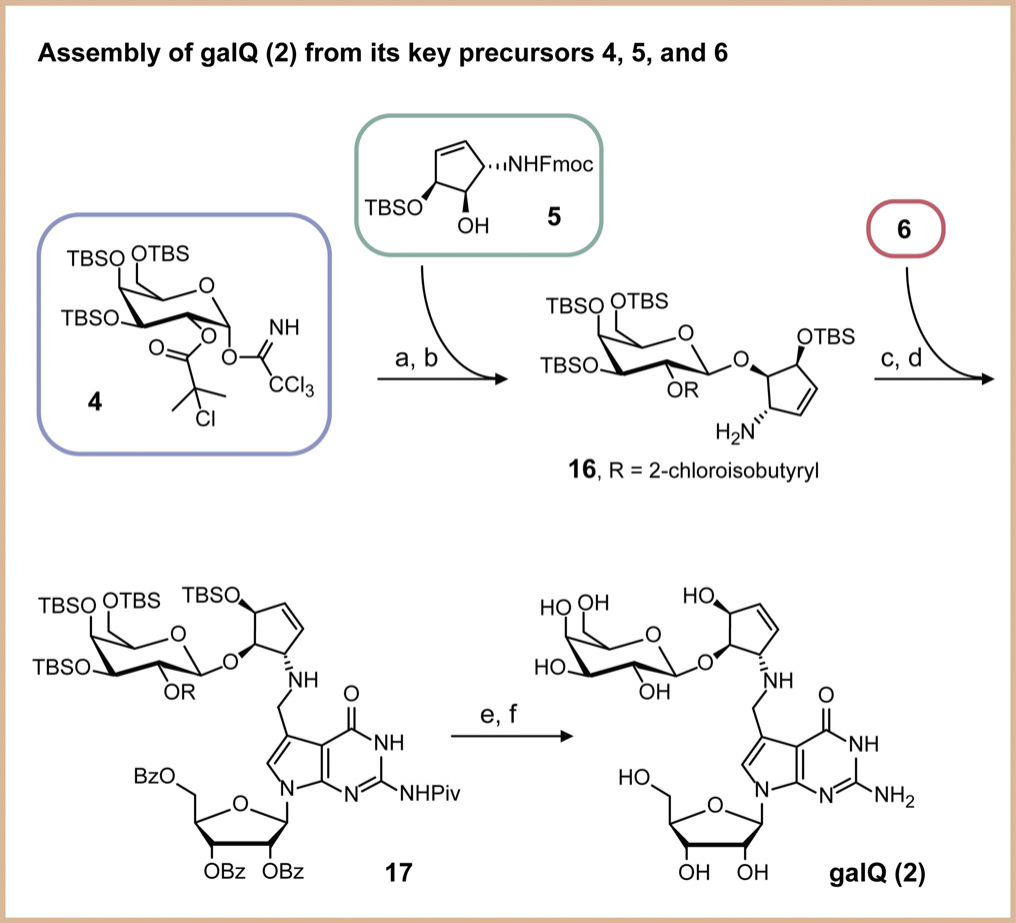
Depiction of the galQ **2** assembly from the three key precursors **4**, **5**, and **6**. a) 2‐Chloro‐6‐methylpyridinium triflate, DCM, rt, 2 h; b) DBU, MeCN, rt, 1.25 h; c) benzene, rt, 5 h; d) NaBH_4_, MeOH, 0 °C, 1 h; e) HF⋅NEt_3_, DCM, rt, 4 d; f) NaOMe, MeOH, rt, 2 d.

We next investigated whether our synthetic β‐homoallylic galQ **2** is identical with the natural product, because analytical data available for galQ was very limited.^11^ For this experiment, we isolated total RNA from mouse liver and performed an enzymatic digestion of the isolated RNA to the nucleoside level. This nucleoside mixture was analyzed by HPLC‐MS. Indeed, under our HPLC conditions, we detected two signals with the appropriate *m*/*z* value for galQ and manQ in the extracted ion chromatogram with a retention time of around 32 and 35 min. No other peaks were present in the same *m*/*z* range. We next co‐injected our synthetic β‐homoallylic galQ **2**, which led to a marked increase of the second signal with a retention time of about 35 min (Figure 3). This result unambigously showed that our synthetic compound galQ **2** and the co‐eluting natural compound with the same mass are identical. Therefore, this natural compound is indeed a β‐galactosylated Q derivative. Taken together, our experiment confirms the proposed chemical structure of galQ, in which the bond between the homoallylic hydroxyl group of queuosine and galactose is in β‐configuration.

**Figure 3:**
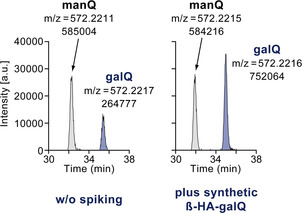
Results of a co‐injection study confirming the identity of our synthetic β‐homoallylic‐galQ **2** and the natural product. Depicted are the extracted‐ion chromatograms (*m*/*z=*572.2148–572.2248) of two HPLC‐MS analyses, either with (right) or without (left) prior spiking of the synthetic galQ **2**. The exact mass [*M*+H]^+^ of galQ (and manQ) is 572.2198 u, showing a perfect match to the two MS peaks observed by us with less than 4 ppm deviation.

Having identified the HPLC retention time of galQ and therefore also of manQ, we finally were able to determine the absolute levels of galQ, manQ, and Q in different tissues of newborn (postnatal day 1; pd1) and adult mice (postnatal month 3; pm3). For an initial broad study, we measured the respective nucleoside levels in cortex, cerebellum, liver, kidney, heart, and spleen, using the same RNA isolation and digestion protocol as for the co‐injection experiment (Figure 4 A).

**Figure 4:**
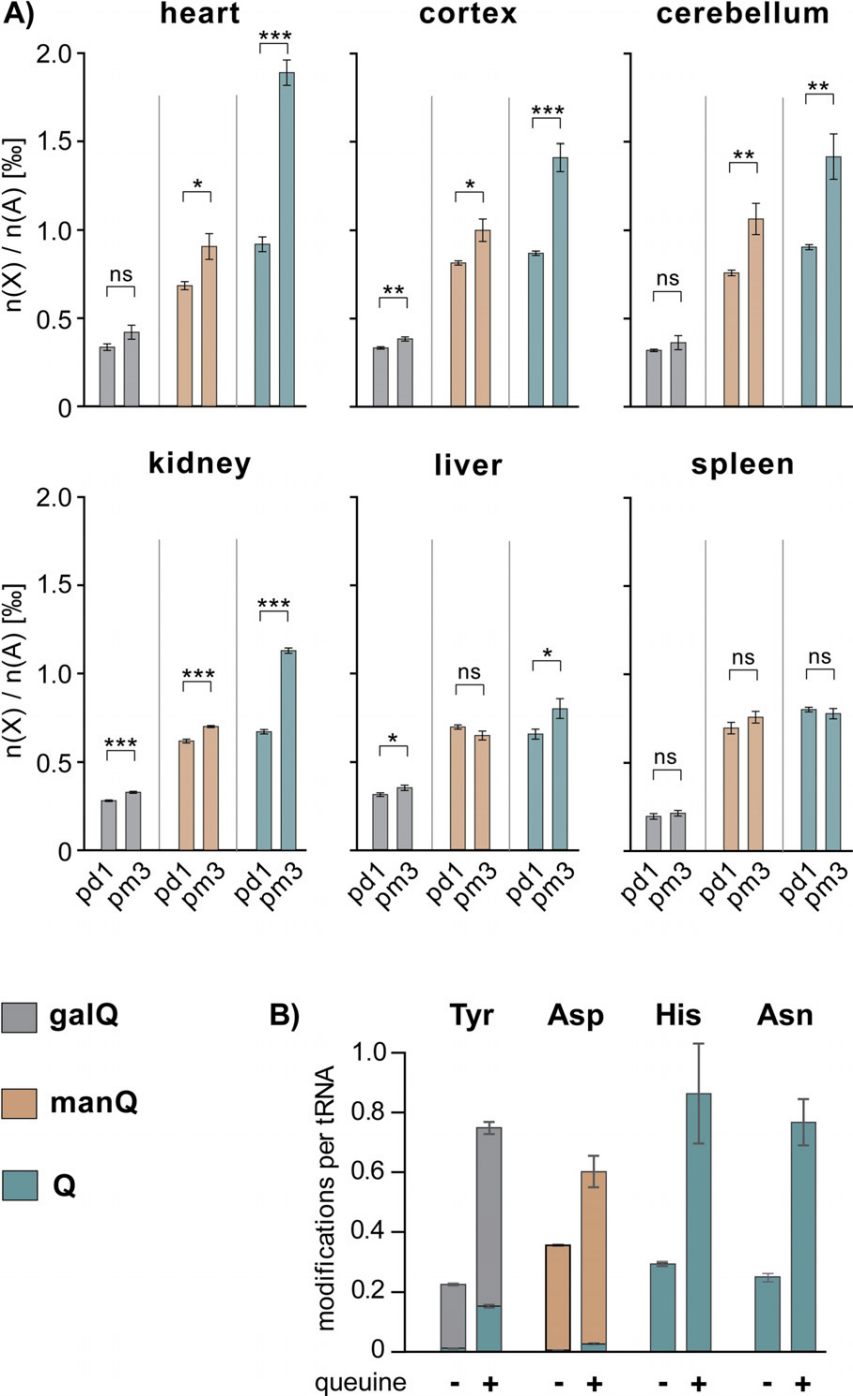
A) Absolute levels of galQ **2**, manQ **3**, and Q **1** in six different organs of newborn (postnatal day 1, pd1) and adult (postnatal month 3, pm3) mice. Values are given as number of xQ modifications n(X) per 1000 adenosine nucleosides n(A). Error bars represent the standard deviation of three biological replicates. For statistical analysis, Student's unpaired two‐tailed t‐test was used. n.s.: not significant, *: *p*<0.05, **: *p*<0.01, ***: *p*<0.001. B) Number of galQ, manQ, and Q modifications per cytosolic tRNA^Tyr^, tRNA^Asp^, tRNA^His^, and tRNA^Asn^ from human HEK 293T cells, respectively. queuine+: cells grown in queuine‐enriched medium, queuine−: cells grown in standard medium. Values are given as average number of modifications per tRNA molecule. Error bars represent the standard deviation of three biological replicates.

From our data it is clearly evident that the levels of all three modifications (galQ, manQ, and Q) generally increase with age. This effect is by far most pronounced with Q, while galQ and manQ only show a modest increase, if at all. Furthermore, and for all three modifications investigated, we see differences between the six organs at the same age. Heart, followed by brain tissues, contains the largest levels of Q and its sugar‐modified derivatives, followed by kidney, liver, and spleen. In general, the changes of the modification levels observed by us positively correlate with the respective organ‐specific protein‐synthesis demands, as we have shown before.^7^ Nevertheless, there are some prominent outliers. These outliers (e.g. heart tissue) seem to rather correlate with the organ‐specific density of mitochondria. It was shown before that the Q‐base in mitochondrial tRNA^Tyr^ and tRNA^Asp^ is not sugar‐modified.^8^ We therefore speculate that the organ‐specific differences in the levels of galQ, manQ, and Q are due to a combination of two independent effects: The organ‐specific protein‐synthesis ratio and the organ‐specific mitochondrial density.

It is well‐established that for biosynthesis of Q (and its sugar‐modified derivatives), eukaryotes have to take up the queuine base from their diet,^21^, ^22^ mammals thereby profiting from their gut microbiome.^23^ We therefore speculate that the low levels of Q‐family nucleosides in newborn mice observed here may be caused by a lack of queuine supply in newborn mice, which only later establish their microbiome. Furthermore, high rates of cell divison and tissue development in young mice may cause additional queuine supply problems.

To further study the influence of queuine availability on Q‐family modification levels, cell culture experiments were performed: Human embryonic kidney cells (HEK 293T) were grown either in culture medium supplemented with 20 nm queuine (enriched medium) or in medium without additional queuine (standard medium). Queuine is the substrate of the TGT enzyme, which performs the exchange of a guanine base by the queuine heterocycle during tRNA maturation.^24^, ^25^, ^26^ From both cell populations, cytosolic *tRNA* ^*Tyr*^, *tRNA* ^*Asp*^, *tRNA* ^*His*^, and *tRNA* ^*Asn*^ were isolated and digested to the nucleoside level. For each of these four individual tRNA species, the number of galQ, manQ, and Q modifications per tRNA was then determined by a mass‐spectrometry‐based isotope‐dilution method using the reference compound synthesized here (see the Supporting Information).

Indeed, our data show that the extent of Q‐modification in the wobble position of cytosolic *tRNA* ^*Tyr*^, *tRNA* ^*Asp*^, *tRNA* ^*His*^, and *tRNA* ^*Asn*^ is strongly dependent on queuine availability (Figure 4 B).^24^ In the case of tRNA^Tyr^ (galQ), tRNA^His^, and tRNA^Asn^ (Q), the difference in modification extent between cells grown in enriched versus standard medium is threefold, while for tRNA^Asp^ (manQ) it is 1.7 fold. These results are well in line with our hypothesis and might therefore explain the lower modification levels in newborn mice. Of note, in all of our experiments even a sufficient queuine supply did not lead to fully modified tRNAs. This might again be an indication of the modification machinery lagging behind the de novo synthesis of tRNA in highly proliferating cells.

Furthermore, we detected a Q‐only‐modified *tRNA* ^*Tyr*^ form in our experiments lacking the galactose sugar, while *tRNA* ^*Asp*^ was always found to be either modified with manQ or completely unmodified. It seems that, in our experimental setup, mannosylation of *tRNA* ^*Asp*^ may be more tightly connected to G/Q‐exchange than the galactosylation of Q‐only‐bearing *tRNA* ^*Tyr*^. Testing this exciting hypothesis is an interesting starting point for future studies.

In summary, we here report the first total synthesis of the human natural product galactosyl‐queuosine **2**. Our synthetic material allowed us to confirm the proposed galQ structure by direct comparison with natural material, and we show that this hypermodfied nucleoside is present in all tissues of newborn and adult mice. We furthermore report the absolute levels of all three Q‐family members in six different mouse organs and in human cytosolic tRNAs. Taken together, our results confirm the crucial importance of tRNA galQ and manQ modification.

## Conflict of interest

The authors declare no conflict of interest.

## Supporting information

As a service to our authors and readers, this journal provides supporting information supplied by the authors. Such materials are peer reviewed and may be re‐organized for online delivery, but are not copy‐edited or typeset. Technical support issues arising from supporting information (other than missing files) should be addressed to the authors.

Supplementary
