# Supplementary material for: Synthesis of Galactosyl‐Queuosine and Distribution of Hypermodified Q‐Nucleosides in Mouse Tissues
Source: Angew Chem Int Ed Engl. 2020 Apr 21;59(30):12352–6. doi: 10.1002/anie.202002295 (PMC7384130; doi:10.1002/anie.202002295)
Supplement: Supplementary file 1 — Supplementary [file ANIE-59-12352-s001.pdf]

Angewandte Chemie
Eine Zeitschrift der Gesellschaft Deutscher Chemiker

Supporting Information

Synthesis of Galactosyl-Queuosine and Distribution of Hypermodified Q-Nucleosides in Mouse Tissues

*Peter Thumbs[+], Timm T. Ensfelder[+], Markus Hillmeier[+], Mirko Wagner[+], Matthias Heiss, Constanze Scheel, Alexander Schön, Markus Müller, Stylianos Michalakis, Stefanie Kellner, and Thomas Carell**

anie_202002295_sm_miscellaneous_information.pdf

Supporting Information

Co-injection of synthetic and natural galQ to confirm its structure 1

Quantification of galQ, manQ, and Q in mouse organs 1

RNA isolation from mouse organs 1

Enzymatic digestion of mouse totalRNA for LC-MS analysis 1

Mass-spectrometry-based quantification of queuosine-family nucleosides in mouse tissues 2

Quantification of galQ, manQ, and Q in human tRNA-isoacceptors 3

Cell culture 3

Cell lysis and tRNA purification 3

Isoacceptor purification 3

Digestion of tRNA isoacceptors for mass spectrometry 4

QQQ mass spectrometry for tRNA isoacceptor analysis 4

Calibration 4

Synthesis of homoallyl-β-GalQ 5

Synthesis of precursor 4 5

3,4,6-Tri-*O*-*tert*-butyldimethylsilyl-D-galactal [4] [18] 6

3,4,6-Tri-*O*-*tert*-butyldimethylsilyl-D-galactopyranose [5] [8] 7

2-Chloroisobutyric acid [6] [19] 8

3,4,6-Tri-*O*-*tert*-butyldimethylsilyl-1,2-di-*O*-(2-chlorisobutyryl)-α-D-galactopyranose [9] 9

3,4,6-Tri-*O*-*tert*-butyldimethylsilyl-2-*O*-(2-chlorisobutyryl)-D-galactopyranose [20] 10

3,4,6-Tri-*O*-*tert*-butyldimethylsilyl-2-*O*-(2-chlorisobutyryl)-α-D-galactopyranosyl-1-*O*-trichloracetimidate [4] 12

Synthesis of precursor 5 13

2,3:5,6-Di-*O*-isopropylidene-α-D-mannofuranose [7] [21] 14

1-*O*-Acetyl-2,3:5,6-Diisopropylidene-α-D-mannofuranose [7] [11] 15

1-*O*-Acetyl-2,3-isopropylidene-α-D-mannofuranose [7] [22] 16

1-*O*-Acetyl-5,6-*O*-methoxymethylen-2,3-*O*-isopropylidene-α-D-mannofuranose [7] [23] 17

(2*S*,3*S*,4*R*)-1-*O*-Acetyl-2,3-*O*-isopropylidene-4-vinyl-α-D-erythrofuranose [7] [12] 18

(2*S*,3*S*,4*R*)-2,3-*O*-Isopropylidene-4-vinylerythrofuranose [7] [24] 19

(3*R*,4*S*,5*R*)-4,5-*O*-Isopropylidenehepta-1,6-dien-3,4,5-triol [7] [13] 20

(3*R*,4*S*,5*R*)-4,5-*O*-Isopropylidenecyclopent-1-en-3,4,5-triol [7] [14] 21

(3*R*,4*S*,5*R*)-3-*O*-Trichloacetimidoyl-4,5-*O*-isopropylidenecyclopent-1-en-3,4,5-triol [8] [25] 22

(3*S*,4*R*,5*S*)-3-Trichloracetylamino-4,5-*O*-isopropylidenecyclopent-1-en-4,5-diol [8] [15] 23

(3*S*,4*R*,5*S*)-(*N*-(9*H*-Fluoren-9-yl)methoxycarbonyl)-4,5-*O*-isopropylidenecyclopent-1-en-4,5-diol [26] 24
(3*S*,4*R*,5*S*)-((*N*-(9*H*-Fluoren-9-yl)methoxycarbonyl))-cyclopent-1-ene-4,5-diol [27]........ 25
(3*S*,4*R*,5*S*)-(*N*-(9*H*-Fluoren-9-yl)methoxycarbonyl)-5-*O*-*tert*-butyldimethylsilylcyclopent-1-en-4,5-diol [5] 26
Synthesis of precursor 6 27
7-Desazaguanine [9] [27] 28
6-Desoxy-6-chloro-7-desazaguanin [9] [28]........ 29
6-Desoxy-6-chloro-2-pivaloylamino-7-desazaguanine [9] [29]........ 30
6-Desoxy-6-chloro-7-iodo-2-pivaloylamino-7-desazaguanine [9] [30]........ 31
2′,3′,5′-Tri-*O*-benzoyl-2-*N*-pivaloyl-6-desoxy-6-chloro-7-iodo-7-desazaguanosine [9] [31] 32
2′,3′,5′-Tri-*O*-benzoyl-2-*N*-pivaloyl-7-iodo-7-desazaguanosin [9] [32] 33
2′,3′,5′-Tri-*O*-benzoyl-2-*N*-pivaloyl-7-formyl-7-desazaguanosine [9] [6] 34
Assembly of GalQ 2 from precursors 4,5 and 6........ 35
1-*O*-((3*S*,4*R*,5*S*)-((*N*-(9*H*-Fluoren-9-yl)methoxycarbonyl)-5-*O*-(*tert*-butyldimethyl-silyl)-cyclopent-1-en-4-yl))-3,4,6-tri-*O*-*tert*-butyldimethylsilyl-2-*O*-(2-chloroiso-butyryl)-β-D-galactopyranose [33] 36
1-*O*-((3*S*,4*R*,5*S*)-(3-Amino-5-*O*-(*tert*-butyldimethylsilyl)cyclopent-1-en-4-yl))-3,4,6-tri-*Otert*-butyldimethylsilyl-2-*O*-(2-chlorisobutyryl)-β-D-galactopyranose [16] 37
7-Desaza-7-((((3*S*,4*R*,5*S*)-4-*O*-(3,4,6-tri-*O*-*tert*-butyldimethylsilyl-2-*O*-(2-chlorisobutyryl)-β-D-galactopyranosyl)-5-*O*-(*tert*-butyldimethylsilyl)cyclopent-1-en-3-yl)amino)-methyl)-2-pivaloylamino-2′,3′,5′-tri-*O*-benzoylguanosin [17]........ 38
7-Desaza-7-((((3*S*,4*R*,5*S*)-4-*O*-(β-D-galactopyranosyl)-5-hydroxy-cyclopent-1-en-3-yl)-amino)methyl)guanosin (galQ) [2] 40
1H-NMR of GalQ (2) 42
^{13}C-NMR of GalQ (2) 44
References........ 46

Co-injection of synthetic and natural galQ to confirm its structure

For this experiment, 5 µg of totalRNA from the livers of two adult mice (postnatal month 3) were digested to the nucleoside level using the *Nucleoside Digestion Mix* from *New England BioLabs* (for details, see *RNA isolation from mouse organs* and *Enzymatic digestion of mouse totalRNA for LC-MS analysis* in this document). 0.5 pmol of our synthetic galactosyl-queuosine (2) were added to the sample which then was analyzed by LC-MS using the same method as described below (see *Mass-spectrometry-based quantification of queuosine-family nucleosides in mouse tissues*).

Quantification of galQ, manQ, and Q in mouse organs

C57BL6/J (Charles River, Sulzfeld, Germany) wildtype mice were used. All procedures concerning animals conform to the German animal protection laws and were approved by the local authority (Regierung von Oberbayern).

RNA isolation from mouse organs

The organs of mice (3 male siblings, 1 day and 3 male siblings, 3 months old) were removed and immediately snap-frozen in liquid nitrogen. For subsequent isolation of totalRNA, 1 mL *TriReagent (Sigma Aldrich)* was used *per* 50 mg organ sample. The tissue was homogenized with a tissue lyzer (*Schwingmühle MM400* from *Retsch*) first at 20 Hz for 4 min, then at 30 Hz for 2 min. The homogenized tissue was transferred into a new 2 mL-tube and mixed with 200 µl chloroform. The phases were separated by centrifugation (12000 g, 15 min, 4°C). The upper clear phase was transferred to a new 2 mL-tube and mixed with 500 µl isopropanol. The RNA was precipitated at -20°C overnight. The precipitated RNA was pelletized by centrifugation (21130 g, 30 min, 4°C). The supernatant was carefully removed and 1 mL of ice-cold 75 % ethanol was added, followed by another centrifugation step (21130 g, 20 min, 4°C). The last three steps were repeated two more times. The supernatant was removed, and the RNA pellet was first dried at room temperature and then dissolved in milliQ-water.

Enzymatic digestion of mouse totalRNA for LC-MS analysis

For each LC-MS-measurement (technical replicate), 3 µg of mouse totalRNA were digested to the nucleoside level using the *Nucleoside Digestion Mix* from *New England BioLabs*. To this reason, a solution of 3 µg totalRNA in 42.5 µL of milliQ-water was prepared. 5 µL of the *Nucleoside Digestion Mix Reaction Buffer (10x)*, and 2.5 µL of the *Nucleoside Digestion Mix* were added, and the mixture was incubated for 2 h at 37 °C. A solution of 0.5 pmol *ß*-allyl-mannosyl-queuosine (internal reference compound) and 1.43 pmol of $^{13}CD_2$-queuosine (heavy-isotope-labeled standard) in 25 µL of milliQ was

added to the sample. The sample was filtered using an *AcroPrep Advance 96 filter plate 0.2 µm Supor* from *Pall Life Sciences* and subsequently analyzed by LC-MS.

Mass-spectrometry-based quantification of queuosine-family nucleosides in mouse tissues

The levels of galactosyl-queuosine (galQ) and mannosyl-queuosine (manQ) in mouse tissues were quantified using a mass spectrometry-based method with synthetic (non-natural) *ß*-allyl-mannosyl-queuosine (amQ) as internal reference compound. To this reason, calibration curves were established, relying on our galQ-compound **2**, but also on synthetic manQ. A detailed manuscript reporting the synthesis of the natural product manQ and of the internal standard *ß*-allyl-mannosyl-Q is currently under preparation. Six calibration solutions were prepared for galQ and manQ (xQ), containing 5.73 to 0.179 pmol galQ and 4.23 to 0.132 pmol manQ in milliQ-water [n(xQ)]. To each of the calibration solutions, 0.80 pmol of *ß*-allyl-mannosyl-Q were added [n(amQ)]. The samples were analyzed by LC-MS, using the same conditions as described below. Ion chromatograms of the compounds of interest were extracted from the total ion current (TIC) chromatograms, and the areas under the curves were integrated. The ratios n(xQ) / n(amQ) were then plotted against the corresponding average peak area ratios A(xQ) / A(amQ) from three independent measurements, and a linerar fit was calculated. The resulting linear equations n(xQ) / n(amQ) = m * [A(xQ) / A(amQ)] + b, with m = 1.8635 and b = 0.0286 for galQ, and m = 1.7328 and b = 0.0234 for manQ, have R^2-values of 0.99 and give backfit-values between 94-102 % (galQ) and 99-105 % (manQ). The equations can be transformed to
n(xQ) = (m * [A(xQ) / A(amQ)] + b) * n(amQ) and were used as calibration curves to calculate the amounts of galQ and manQ [n(xQ)] the mouse tissue samples.
Levels of queuosine and adenosine were quantified in parallel with galQ and manQ. For queuosine quantification, $^{13}CD_2$-Q was used as heavy-atom-labeled internal standard, while adenosine was quantified based on its UV-absorption. The corresponding calibration curves were published by us before.

Quantitative HPLC-HESI-MS analysis of the enzymatically digested RNA samples was performed on a *Dionex Ultimate 3000 HPLC system* coupled to a *Thermo Fisher LTQ Orbitrap XL* mass spectrometer with an injection volume of 65 µL (of 75 µL total sample volume) per measurement. Nucleosides were separated on an *Interchim Uptisphere120-3HDO C18* column whose temperature was maintained at 30 °C. Elution buffers were buffer A (2 mM NH_4HCOO in H_2O; pH 5.5) and buffer B (2 mM NH_4HCOO in H_2O/MeCN 20/80 v/v; pH 5.5) with a flow rate of 0.15 mL/min. The gradient was as follows: 0→10 min, 0% B; 10→50 min, 0→5 % B; 50→75 min, 5→60 % B; 75→80 min, 60→100 % B. The chromatogram was recorded at 260 nm with a *Dionex Ultimate 3000 Diode Array Detector*, and the chromatographic eluent was directly injected into the ion source of the mass spectrometer without prior splitting. Ions were scanned in the positive polarity mode over a full-scan range of m/z = 225-800 with a resolution of 60,000. Parameters of the mass spectrometer were tuned with a freshly mixed solution of inosine (5 µM) in buffer A and set as follows: Capillary temperature 275.00 °C; source voltage 4.80 kV; vaporizer temperature 100 °C, capillary voltage 0 V; tube lens voltage 45.00 V. The ion chromatograms of the compounds of interest were extracted from the total ion current (TIC)

chromatogram, and the areas under the curves were integrated. The amount of galQ, manQ, and Q in a sample was calculated using the respective calibration curves. For each of the three biological replicates per tissue and age, two independent measurements (technical replicates) were performed. Resulting values are presented as n(X) / n(A).

Quantification of galQ, manQ, and Q in human tRNA-isoacceptors

Cell culture

Cell culture media and supplements were obtained from *Sigma-Aldrich* (Munich, Germany) unless stated otherwise. Queuine base was obtained from *TRC* (North York, Canada). Standard Basal medium for HEK-293T culture was DMEM D6546 high glucose supplemented with 10 % FBS and 0.584 g/L L-glutamine. Queuine base was supplemented at a concentration of 20 nM. Cells were split 1:7 using standard procedures every 2-3 days to counter overgrowth. Cells were kept at 10 % CO_2 for proper pH-adjustment. For biological replicates, one culture was split into several flasks prior to harvesting.

Cell lysis and tRNA purification

Cells were directly harvested on T25 flasks (*TPP*, Trasadingen, Swiss) using 1 mL TRI reagent. totalRNA was isolated according to the supplier's manual. tRNA was purified by size exclusion chromatography (SEC) and ethanol precipitation according to published procedures.[1] [2] The tRNA was resuspended in water (35 µL).

Isoacceptor purification

The procedure was adapted from Hauenschild *et al.*.[3] For isoacceptor purification, 1 µg pre-purified total tRNA was used. The sequences of the biotinylated 2'-deoxyoligonucleotides are:

$tRNA^{Asp}_{GUC}$:

5'-[Biotin]TGGCTCCCCGTCGGGGAATTGAACCCCGGTCTCCCGCGTGACAGGCGGGGATACTAACCACTATACTAACGAGGAAAA-3'

$tRNA^{His}_{GUG}$:

5'-[Biotin]TGCCGTCACTCGGATTCGAACCGAGGTTGCTG-3'

$tRNA^{Asn}_{GUU}$:

5'-[Biotin]AAATGGCGTCCCTGGGTGGGCTCGAACCACCAACCTTTTCGGTTAACAGCC-3'

$tRNA^{Tyr}_{GUA}$:

5'-[Biotin]AAATGGTCCTTCGAGCCGGAATCGATCCAGCGA-3'

Digestion of tRNA isoacceptors for mass spectrometry

tRNA isoacceptors in aqueous digestion mix (20 µL) were digested to single nucleosides by using 2 U alkaline phosphatase, 0.2 U phosphodiesterase I (*VWR*, Radnor, Pennsylvania, USA), and 2 U benzonase in Tris (pH 8, 5 mM) and $MgCl_2$ (1 mM) containing buffer. Furthermore, 0.5 µg tetrahydrouridine (*Merck*, Darmstadt, Germany), 1 µM butylated hydroxytoluene, and 0.1 µg pentostatin were added to avoid deamination and oxidation of the nucleosides. After incubation for 2 h at 37 °C, 10 µL of LC-MS running buffer was added to the mixture and then filtered through 96-well filter plates (*AcroPrep Advance 350 10K Omega, PALL Corporation*, New York, USA) at 3000 g and 4 °C for 30 min. 1/10 vol. of SILIS (stable isotope labeled internal standard), as prepared in Heiss *et al.*, was added to each filtrate before analysis by QQQ-mass spectrometry.[2] For each sample 10 µL were injected.

QQQ mass spectrometry for tRNA isoacceptor analysis

For quantitative mass spectrometry, an *Agilent 1290 Infinity II* equipped with a diode-array detector (DAD) combined with an *Agilent Technologies G6470A Triple Quad* system and electrospray ionization (ESI-MS, *Agilent Jetstream*) was used. Operating parameters: positive-ion mode, skimmer voltage of 15 V, cell accelerator voltage of 5 V, N_2 gas temperature of 230 °C and N_2 gas flow of 6 L/min, sheath gas (N_2) temperature of 400 °C with a flow of 12 L/min, capillary voltage of 2500 V, nozzle voltage of 0 V, and nebulizer at 40 psi. The instrument was operated in dynamic MRM mode. For separation a *Synergi, 2.5 µm Fusion-RP* C_{18}*, 100 Å, 100 x 2 mm* column (*Phenomenex®, Torrance*, California, USA) at 35 °C and a flow rate of 0.35 mL/min was used in combination with a binary mobile phase of 5 mM NH_4OAc aqueous buffer A, brought to pH 5.6 with glacial acetic acid (65 µL / L buffer), and an organic buffer B of pure acetonitrile (*Roth*, Ultra LC-MS grade, purity ≥99.98). The gradient started at 100 % solvent A, followed by an increase to 10 % over 6 min. From 10 to 15 min, solvent B was increased to 45 % and was maintained for 3 min before returning to 10 % solvent A and a 3 min re-equilibration period.

Calibration

For calibration, synthetic nucleosides were weighed and dissolved in water to a stock concentration of 1-10 mM. The nucleosides adenosine (A), cytidine (C), guanosine (G) and uridine (U), were obtained from *Sigma-Aldrich*. Queuosine (Q) was a generous gift from the *Dedon* lab, manQ and galQ were provided by the *Carell* lab. The calibration solutions range from 0.025 to 100 pmol for each canonical nucleoside and from 0.25 fmol to 1 pmol for Q-modifications. Each calibration was spiked with 10 % SILIS. The sample data were analyzed by the *Quantitative MassHunter* Software from *Agilent*. The absolute amounts of the modifications were referenced to the absolute amounts of injected tRNA (based on expected and calculated amount of canonical nucleosides).

Synthesis of homoallyl-β-GalQ

Synthesis of precursor 4

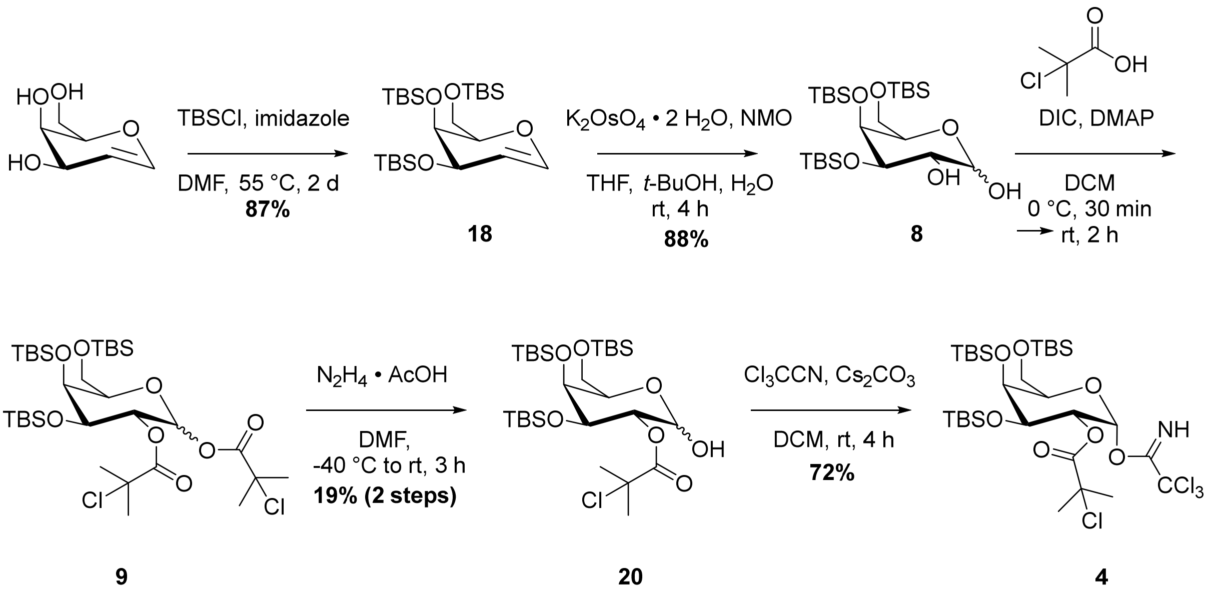

3,4,6-Tri-*O*-*tert*-butyldimethylsilyl-D-galactal [4] [18]

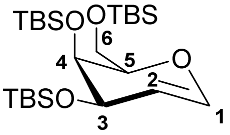

D-(+)-Galactal **6** (1.47 g, 10.1 mmol, 1.0 eq.) was dissolved in DMF (28 mL) and imidazole (4.82 g, 70.8 mmol, 7.00 eq.) and TBSCl (5.23 g, 34.7 mmol, 3.5 eq.) were added. The solution was stirred for 2 d at 55 °C and then poured into water (300 mL). The aqueous phase was extracted with diethyl ether (3 x 150 mL). The combined organic phases were washed with water (300 mL), dried over $MgSO_4$ and the solvent was removed *in vacuo*. Column chromatographic purification (Isohexane/EtOAc 200:1 → 150:1) gave the product (4.29 g, 8.77 mmol, 87%) as colorless oil.

TLC [Isohexane/EtOAc (8:1)]: R_f = 0.75.

^{1}H-NMR (400 MHz, $CDCl_3$, 27 °C): δ = 6.21 (dd, $^3J_{H1,H2}$ = 6.1 Hz, $^4J_{H1,H3}$ = 0.8 Hz, 1H; C1H), 4.68 – 4.62 (m, 1H; C2H), 4.16 – 3.98 (m, 4H; C3H, C4H, C5H, C6Ha), 3.90 – 3.85 (m, 1H; C6Hb), 0.91 (s, 9H; SiC(CH_3)$_3$), 0.90 (2s, 18H; SiC(CH_3)$_3$), 0.10 (2s, 6H; $SiCH_3$), 0.07 (2s, 6H; $SiCH_3$), 0.07 (s, 3H; $SiCH_3$), 0.06 (s, 3H; $SiCH_3$) ppm.

^{13}C-NMR (100 MHz, $CDCl_3$, 27 °C): δ = 142.7 (C1), 102.6 (C2), 79.6 (C5), 68.7 (C4), 65.0 (C3), 60.9 (C6), 26.0 (2 × 3C; $SiC(CH_3)_3$), 25.9 (3C; $SiC(CH_3)_3$), 18.5 ($SiC(CH_3)_3$), 18.3 ($SiC(CH_3)_3$), 18.2 ($SiC(CH_3)_3$), −4.2 ($SiCH_3$), −4.4 ($SiCH_3$), −4.8 ($SiCH_3$), −4.9 ($SiCH_3$), −5.1 ($SiCH_3$), −5.3 ($SiCH_3$) ppm.

HRMS (ESI+) *m/z*: calc. for $[C_{24}H_{52}O_4Si_3 + NH_4]^+$: 506.3512, found: 506.3515.

3,4,6-Tri-*O*-*tert*-butyldimethylsilyl-D-galactopyranose [5] [8]

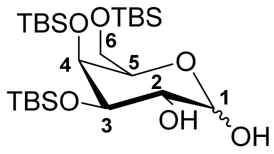

3,4,6-Tri-*O*-tert-butyldimethylsilyl-D-galactal (2.09 g, 4.27 mmol, 1.0 eq.) was dissolved in a mixture of THF (14 mL), tert-butanol (6 mL) and water (2 mL). NMO (1.51 g, 12.9 mmol, 3.0 eq.) and K_2OsO_4·2 H_2O (70.0 mg, 200 µmol, 0.05 eq.) were added and the reaction mixture was stirred for 48 h at room temperature. The solution was diluted with water (40 mL) and Na_2SO_3 (2.69 g, 21.4 mmol, 5.00 eq) was added and the mixture was stirred for 2 h at room temperature. Water (40 mL) was added and the aqueous suspension was extracted with EtOAc (3 x 100 mL). The combined organic phases were washed with brine, dried over $MgSO_4$, filtered and the solvent was removed in vacuo. Column chromatographic purification (Isohexane/EtOAc 9:1) gave an anomeric mixture of the product (1.96 g, 3.75 mmol, 88%, α/β ca. 2.5:1)

TLC [Isohexane/EtOAc (8:1)]: R_f = 0.12 (α), R_f = 0.14 (β).

α-Anomer:

^{1}H-NMR (600 MHz, $CDCl_3$, 27 °C): δ = 5.29 (d, $^3J_{H1,H2}$ = 3.6 Hz, 1H; C1H), 4.01 – 3.98 (m, 1H; C4H), 3.92 (dd, $^3J_{H2,H3}$ = 9.5 Hz, $^3J_{H2,H1}$ = 3.6 Hz, 1H; C2H), 3.88 (t, $^3J_{H5,H6a}$ = $^3J_{H5,H6b}$ = 6.7 Hz, 1H; C5H), 3.87 (dd, $^3J_{H3,H2}$ = 9.6 Hz, $^3J_{H3,H4}$ = 2.3 Hz, 1H; C3H), 3.69 – 3.63 (m, 2H; C6Ha, C6Hb), 0.94 (s, 9H; $SiC(CH_3)_3$), 0.90 (s, 9H; $SiC(CH_3)_3$), 0.89 (s, 9H; $SiC(CH_3)_3$), 0.14 (s, 3H; $SiCH_3$), 0.13 (s, 3H; $SiCH_3$), 0.12 (s, 3H; $SiCH_3$), 0.08 (s, 3H; $SiCH_3$), 0.06 (2s, 6H; $SiCH_3$) ppm.

13C-NMR (150 MHz, $CDCl_3$, 27 °C): δ = 93.0 (C1), 72.7 (2C; C3, C5), 71.5 (C4), 69.5 (C2), 61.7 (C6), 26.3 (3C; $SiC(CH_3)_3$), 26.1 (3C; $SiC(CH_3)_3$), 25.9 (3C; $SiC(CH_3)_3$), 18.6 ($SiC(CH_3)_3$), 18.5 ($SiC(CH_3)_3$), 18.2 ($SiC(CH_3)_3$), −3.9 (2C; $SiCH_3$), −4.4 ($SiCH_3$), −4.8 ($SiCH_3$), −5.3 (2C; $SiCH_3$) ppm.

β-Anomer:

^{1}H-NMR (600 MHz, $CDCl_3$, 27 °C): δ = 4.53 (d, $^3J_{H1,H2}$ = 7.3 Hz, 1H; C1H), 3.95 (d, $^3J_{H4,H3}$ = 2.1 Hz, 1H; C4H), 3.72 (dd, $^2J_{H6a,H6b}$ = 10.0 Hz, $^3J_{H6a,H5}$ = 7.8 Hz, 1H; C6Ha), 3.69 – 3.63 (m, 2H; C6Hb, C2H), 3.53 (dd, $^3J_{H3,H2}$ = 9.6 Hz, $^3J_{H3,H4}$ = 2.4 Hz, 1H; C3H), 3.41 (ddd, $^3J_{H5,H6a}$ = 7.7 Hz, $^3J_{H5,H6b}$ = 5.8 Hz, $^3J_{H5,H4}$ = 0.6 Hz, 1H; C5H), 0.94 (s, 9H; $SiC(CH_3)_3$), 0.90 (s, 9H; $SiC(CH_3)_3$), 0.89 (s, 9H; $SiC(CH_3)_3$), 0.14 (2s, 6H; $SiCH_3$), 0.13 (s, 3H; $SiCH_3$), 0.09 (s, 3H; $SiCH_3$), 0.06 (2s, 6H; $SiCH_3$) ppm.

^{13}C-NMR (150 MHz, $CDCl_3$, 27 °C): δ = 97.6 (C1), 76.2 (C5), 75.9 (C3), 72.9 (C2), 70.7 (C4), 61.3 (C6), 26.3 (3C; $SiC(CH_3)_3$), 26.1 (3C; $SiC(CH_3)_3$), 25.8 (3C; $SiC(CH_3)_3$), 18.6 (2C; $SiC(CH_3)_3$), 18.2 ($SiC(CH_3)_3$), −3.9 (2C; $SiCH_3$), −4.4 ($SiCH_3$), −4.7 ($SiCH_3$), −5.3 (2C; $SiCH_3$) ppm.

HRMS (ESI+) *m/z*: calc. for [$C_{24}H_{54}O_6Si_3$ + Na]$^+$: 545.3120, found: 545.3122.

2-Chloroisobutyric acid [6] [19]

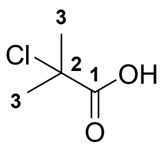

Isobutyric acid (9.30 mL, 100 mmol), *N*-Chlorosuccinimide (10.0 g, 74.9 mmol, 0.75 eq.) and concentrated sulfuric acid (2.51 g) were dissolved in TFA (50 mL) and stirred at 100 °C for 2 d. Another portion of *N*-Chlorsuccinimide (10.0 g, 74.9 mmol, 0.75 eq.) was added and stirring continued at 100 °C for 2 d. Addtion of more *N*-Chlorsuccinimide (10.1 g, 75.4 mmol, 0.75 eq.) led to completion of the reaction after stirring for another 24 h at 100 °C. The product was purified by fractionated vacuum distillation (130 °C, 10 – 15 Pa), which gave a a crude product. Another distillation step (60 °C, 10 – 15 Pa) gave the product (5.22 g 42.6 mmol, 42%) as colorless oil, which crystallized after cooling to colorless needles.

^{1}H-NMR (400 MHz, D_2O, 27 °C): δ = 1.80 (s, 6H; C3H) ppm.

^{13}C-NMR (100 MHz, D_2O, 27 °C): δ = 175.8 (C1), 66.0 (C2), 29.0 (C3) ppm.

HRMS (EI) *m/z*: calc. for [$C_4H_7O_2$]: 122.0135, found: 122.0116.

3,4,6-Tri-*O*-*tert*-butyldimethylsilyl-1,2-di-*O*-(2-chlorisobutyryl)-α-D-galactopyranose [9]

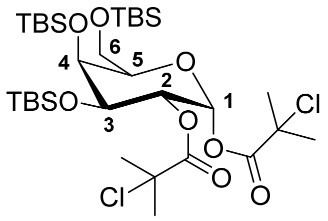

3,4,6-Tri-*O*-*tert*-butyldimethylsilyl-D-galactopyranose (5.03 g, 9.62 mmol, 1.0 eq.) was dissolved in DCM (68 mL) and a solution of 2-chloroisobutyric acid (2.85 g, 23.3 mmol, 2.40 eq.) in DCM (20 mL) and DMAP (70.0 mg, 600 µmol, 0.05 eq.) were added. Subsequently DIC (3.75 mL, 24.1 mmol, 2.50 eq.) was added at 0 °C and the solution was stirred for 30 min at 0 °C and then for 2 h at room temperature. The precipitate was filtered off and the organic phase was washed with saturated aqueous $NaHCO_3$-solution. The aqueous phase was re-extracted with diethyl ether (3 x 60 mL) and the combined organic phases were dried over $MgSO_4$, filtered and the solvent was removed *in vacuo*. Column chromatographic purification (Isohexan/EtOAc 100:1) gave a mixture of the product (4.17 g) with the resulting urea derivative as colorless oil.

TLC [Isohexane/EtOAc (8:1)]: R*f* = 0.56.

^{1}H-NMR (600 MHz, $CDCl_3$, 27 °C): δ = 6.29 (d, $^3J_{H1,H2}$ = 3.4 Hz, 1H; C1H), 5.46 (dd, $^3J_{H2,H3}$ = 10.3 Hz, $^3J_{H2,H1}$ = 3.4 Hz, 1H; C2H), 4.21 (dd, $^3J_{H3,H2}$ = 10.3 Hz, $^3J_{H3,H4}$ = 2.2 Hz, 1H; C3H), 4.14 (d, $^3J_{H4,H3}$ = 2.3 Hz, 1H; C4H), 3.88 (t, $^3J_{H5,H6a}$ = $^3J_{H5,H6b}$ = 6.8 Hz, 1H; C5H), 3.70 (dd, $^3J_{H6a,H6b}$ = 10.3 Hz, $^3J_{H6a,H5}$ = 7.6 Hz, 1H; C6Ha), 3.64 (dd, $^3J_{H6b,H6a}$ = 10.3 Hz, $_3J_{H6b,H5}$ = 6.1 Hz, 1H; C6H), 1.81 (s, 3H; $CCl(CH_3)_2$), 1.79 (s, 3H; $CCl(CH_3)_2$), 1.76 (s, 3H; $CCl(CH_3)_2$), 1.73 (s, 3H; $CCl(CH_3)_2$), 0.93 (s, 9H; $SiC(CH_3)_3$), 0.92 (s, 9H; $SiC(CH_3)_3$), 0.87 (s, 9H; $SiC(CH_3)_3$), 0.17 (s, 3H; $SiCH_3$), 0.16 (s, 6H; $SiCH_3$), 0.11 (s, 3H; $SiCH_3$), 0.04 (s, 3H; $SiCH_3$), 0.03 (s, 3H; $SiCH_3$) ppm.

^{13}C-NMR (150 MHz, $CDCl_3$, 27 °C): δ = 171.3 ($COCCl(CH_3)_2$), 169.8 ($COCCl(CH_3)_2$), 91.7 (C1), 75.3 (C5), 71.8 (C4), 71.5 (C2), 70.0 (C3), 64.5 ($CCl(CH_3)_2$), 64.3 ($CCl(CH_3)_2$), 61.0 (C6), 30.0 (2C; $CCl(CH_3)_2$), 29.7 ($CCl(CH_3)_2$), 29.6 ($CCl(CH_3)_2$), 26.2 (3C; $SiC(CH_3)_3$), 26.0 (3C; $SiC(CH_3)_3$), 25.7 (3C; $SiC(CH_3)_3$), 18.6 ($SiC(CH_3)_3$), 18.3 ($SiC(CH_3)_3$), 18.1 ($SiC(CH_3)_3$), −3.8 ($SiCH_3$), −3.9 ($SiCH_3$), −4.7 ($SiCH_3$), −4.9 ($SiCH_3$), −5.3 ($SiCH_3$), −5.4 ($SiCH_3$) ppm.

HRMS (ESI+) m/z: calc. for $[C_{32}H_{64}O_8Cl_2Si_3 + NH_4]^+$: 748.3624, found: 748.3630.

3,4,6-Tri-*O*-*tert*-butyldimethylsilyl-2-*O*-(2-chlorisobutyryl)-D-galactopyranose [20]

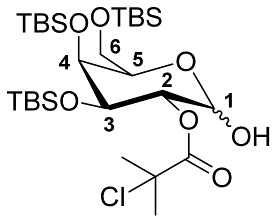

A mixture of 3,4,6-Tri-*O*-*tert*-butyldimethylsilyl-1,2-di-*O*-(2-chlorisobutyryl)-α-D-galactopyranose and the urea derivative (4.14 g) was dissolved in DMF (40 mL) and cooled to −40 °C. N_2H_4·AcOH (0.42 g, 4.56 mmol) was added at that temperature and the solution was allowed to warm to room temperature over a period of 30 min. Subsequently the solution was stirred for 3 h at room temperature. The solution was poured into water (400 mL) and the suspension was extracted with diethyl ether (3 x 120 mL). The combined organic phases were washed with water (100 mL), dried over $MgSO_4$, filtered and the solvent was removed in vacuo. Column chromatographic purification (Isohexane/EtOAc 50:1 → 9:1) gave an anomeric mixture of the product (1.14 g, 1.82 mmol, 19% over 2 steps) as colorless foam.

TLC [Isohexane/EtOAc (8:1)]: R*f* = 0.27 (α und β).

α-Anomer:
^{1}H-NMR (300 MHz, $CDCl_3$, 27 °C): δ = 5.38 (d, $^{3}J_{H1,H2}$ = 3.3 Hz, 1H; C1H), 5.20 – 5.12 (m, 1H; C2H), 4.15 (dd, $^{3}J_{H3,H4}$ = 9.4 Hz, $^{3}J_{H3,H2}$ = 2.3 Hz, 1H; C3H), 4.10 – 4.05 (m, 1H; C4H), 3.94 (td, $^{3}J_{H5,H6a}$ = $^{3}J_{H5,H6b}$ = 6.7 Hz, 3$J_{H5,H4}$ = 1.1 Hz, 1H; C5H), 3.80 – 3.65 (m, 2H; C6Ha, C6Hb), 2.65 (bs, 1H; C1-OH), 1.81 (2s, 6H; $CCl(CH_3)_2$), 0.93 (s, 9H; $SiC(CH_3)_3$), 0.91 (s, 9H; $SiC(CH_3)_3$), 0.89 (s, 9H; $SiC(CH_3)_3$), 0.18 (s, 3H; $SiCH_3$), 0.16 (s, 3H; $SiCH_3$), 0.14 (s, 3H; $SiCH_3$), 0.12 (s, 3H; $SiCH_3$), 0.06 (s, 6H; $SiCH_3$) ppm.

^{13}C-NMR (75 MHz, $CDCl_3$, 27 °C): δ = 171.4 ($COCCl(CH_3)_2$), 96.2 (C1), 73.7 (C2), 73.3 (C5), 71.5 (C4), 69.6 (C3), 64.6 ($CCl(CH_3)_2$), 60.9 (C6), 29.9 (2C; $CCl(CH_3)_2$), 26.2 (2 × 3C; $SiC(CH_3)_3$), 25.8 (3C; $SiC(CH_3)_3$), 18.5 ($SiC(CH_3)_3$), 18.2 (2C; $SiC(CH_3)_3$), −3.8 (2C; $SiCH_3$), −4.6 ($SiCH_3$), −4.8 ($SiCH_3$), −5.3 (2C; $SiCH_3$) ppm.

β-Anomer:
^{1}H-NMR (300 MHz, $CDCl_3$, 27 °C): δ = 5.20 – 5.12 (m, 1H; C2H), 4.58 (bs, 1H; C1H), 4.10 – 4.05 (m, 1H; C4H), 3.80 – 3.65 (m, 3H; C3H, C6Ha, C6Hb), 3.49 – 3.43 (m, 1H; C5H), 3.05 (bs, 1H; C1-OH), 1.80 (2s, 6H; $CCl(CH_3)_2$), 0.92 (2s, 18H; $SiC(CH_3)_3$), 0.89 (s, 9H; $SiC(CH_3)_3$), 0.15 (s, 3H; $SiCH_3$), 0.14 (s, 3H; $SiCH_3$), 0.13 (s, 3H; $SiCH_3$), 0.10 (s, 3H; $SiCH_3$), 0.06 (s, 6H; $SiCH_3$) ppm.

^{13}C-NMR (75 MHz, $CDCl_3$, 27 °C): δ = 172.2 ($COCCl(CH_3)_2$), 90.2 (C1), 76.5 (C2), 76.2 (C5), 73.3 (C3), 70.8 (C4), 64.5 ($CCl(CH_3)_2$), 61.0 (C6), 30.2 ($CCl(CH_3)_2$), 30.1 ($CCl(CH_3)_2$), 26.2 (3C; $SiC(CH_3)_3$), 26.1

(3C; $SiC(CH_3)_3$), 25.8 (3C; $SiC(CH_3)_3$), 18.6 ($SiC(CH_3)_3$), 18.2 ($SiC(CH_3)_3$), 18.1 ($SiC(CH_3)_3$), −3.8 ($SiCH_3$), −4.0 ($SiCH_3$), −4.6 (2C; $SiCH_3$), −5.2 ($SiCH_3$), −5.3 ($SiCH_3$) ppm.

HRMS (ESI+) *m/z*: calc. for $[C_{28}H_{59}O_7ClSi_3 + Na]^+$: 649.3149, found: 649.3151.

3,4,6-Tri-*O*-*tert*-butyldimethylsilyl-2-*O*-(2-chlorisobutyryl)-α-D-galactopyranosyl-1-*O*-trichloracetimidate [4]

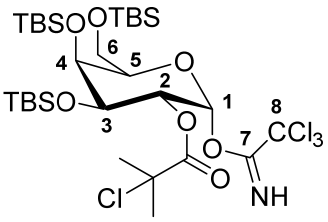

To a solution of 3,4,6-Tri-*O*-*tert*-butyldimethylsilyl-2-*O*-(2-chlorisobutyryl)-D-galactopyranose (2.50 g, 3.98 mmol, 1.0 eq.) in DCM (125 mL) Cs_2CO_3 (780 mg, 2.40 mmol, 0.60 eq.) and Cl_3CCN (4.00 mL, 39.9 mmol, 10.0 eq.) were added. The solution was stirred at room temperature for 4 h. Subsequently saturated aqueous $NaHCO_3$-solution (100 mL) was added and the mixture was stirred for 5 min at room temperature. The phases were separated and the organic phase was extracted with DCM (4 x 100 mL). The combined organic phases were dried over $MgSO_4$ and the solvent was removed *in vacuo*. Column chromatographic purification (Isohexane/EtOAc 100:1) gave the product (2.21 g, 2.86 mmol, 72%, only α) as colorless oil.

TLC [Isohexane/EtOAc (8:1)]: R*f* = 0.50.

^{1}H-NMR (600 MHz, $CDCl_3$, 27 °C): δ = 8.54 (s, 1H; $NHCOCl_3$), 6.48 (d, $^3J_{H1,H2}$ = 3.4 Hz, 1H; C1H), 5.49 (dd, $^3J_{H2,H3}$ = 10.4 Hz, $^3J_{H2,H1}$ = 3.4 Hz, 1H; C2H), 4.30 (dd, $^3J_{H3,H2}$ = 10.4 Hz, $^3J_{H3,H4}$ = 2.2 Hz, 1H; C3H), 4.16 (d, $^3J_{H4,H3}$ = 1.9 Hz, 1H; C4H), 3.94 (dd, $^3J_{H5,H6a}$ = 7.8 Hz, $^3J_{H5,H6b}$ = 6.1 Hz, 1H; C5H), 3.72 (dd, $^2J_{H6a,H6b}$ = 10.2 Hz, $_3J_{H6a,H5}$ = 8.0 Hz, 1H; C6Ha), 3.64 (dd, $^2J_{H6b,H6a}$ = 10.2 Hz, $3J_{H6b,H5}$ = 5.9 Hz, 1H; C6Hb), 1.76 (s, 3H; $CCl(CH_3)_2$), 1.74 (s, 3H; $CCl(CH_3)_2$), 0.93 (s, 9H; $SiC(CH_3)_3$), 0.92 (s, 9H; $SiC(CH_3)_3$), 0.86 (s, 9H; $SiC(CH_3)_3$), 0.18 (s, 3H; $SiCH_3$), 0.16 (s, 3H; $SiCH_3$), 0.15 (s, 3H; $SiCH_3$), 0.12 (s, 3H; $SiCH_3$), 0.02 (s, 3H; $SiCH_3$), 0.01 (s, 3H; $SiCH_3$) ppm.

^{13}C-NMR (150 MHz, $CDCl_3$, 27 °C): δ = 171.4 ($COCCl(CH_3)_2$), 160.9 ($NHCOCl_3$), 94.3 (C1), 91.4 (CCl_3), 75.1 (C5), 71.7 (2C; C2, C4), 69.9 (C3), 64.2 ($CCl(CH_3)_2$), 60.8 (C6), 30.0 (2C; $CCl(CH_3)_2$), 26.2 (3C; $SiC(CH_3)_3$), 26.0 (3C; $SiC(CH_3)_3$), 25.7 (3C; $SiC(CH_3)_3$), 18.6 ($SiC(CH_3)_3$), 18.3 ($SiC(CH_3)_3$), 18.1 ($SiC(CH_3)_3$), −3.8 ($SiCH_3$), −4.0 ($SiCH_3$), −4.7 ($SiCH_3$), −4.9 ($SiCH_3$), −5.3 ($SiCH_3$), −5.4 ($SiCH_3$) ppm.
HRMS (ESI−) *m/z*: calc. for $[C_{30}H_{59}NO_7Cl_4Si_3 + Cl]^-$: 804.2047, found: 804.2064.

Synthesis of precursor 5

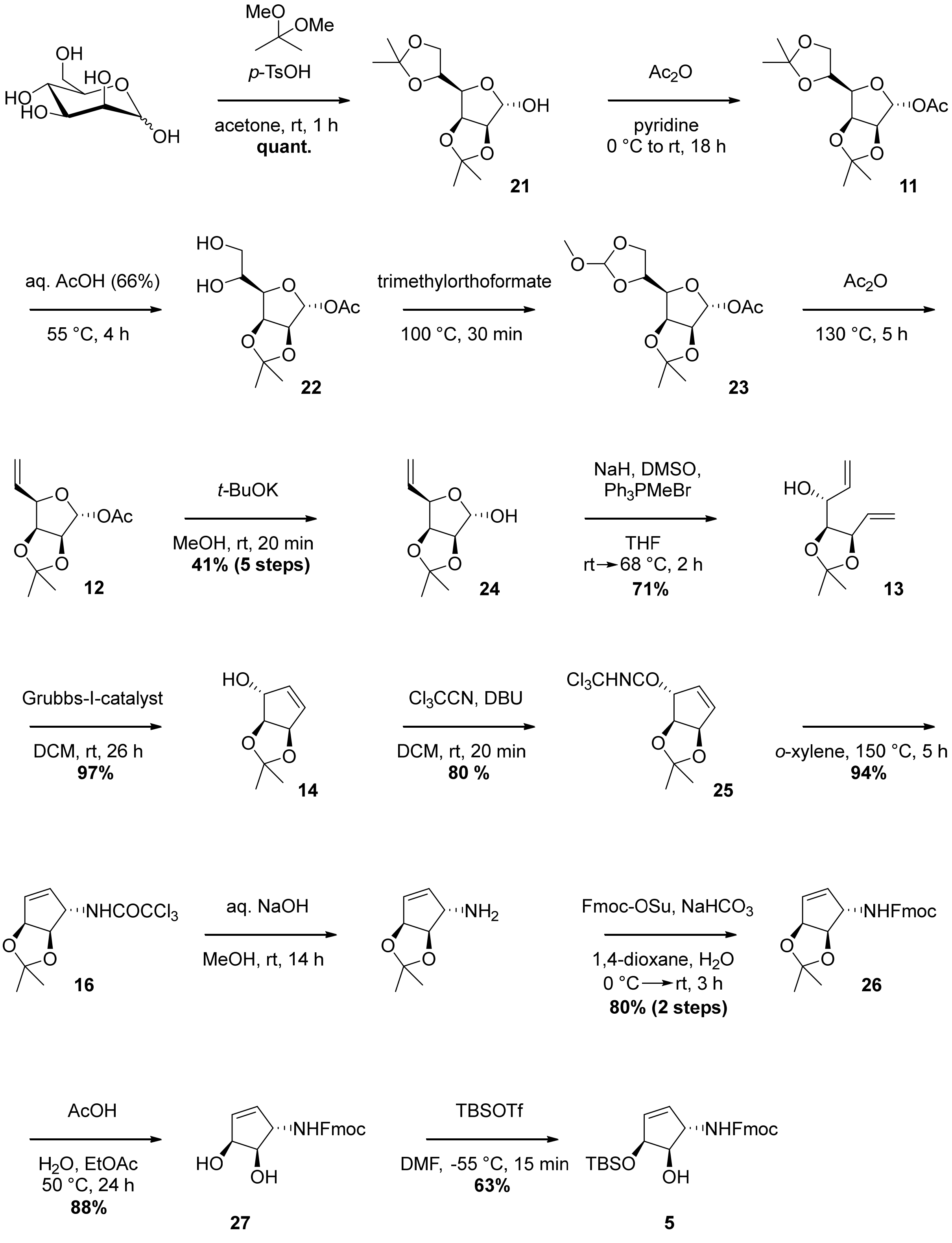

2,3:5,6-Di-*O*-isopropylidene-α-D-mannofuranose [7] [21]

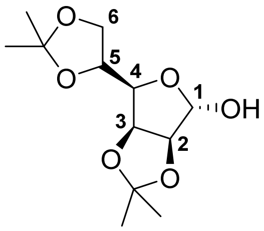

D-Mannose (54.0 g, 300 mmol, 1.0 eq.) was suspended in 2,2-dimethoxypropane (160 mL, 887 mmol, 4.4 eq.) and acetone (160 mL) and a catalytic amount of *p*-toluene sulfonic acid (5.70 g, 30 mmol, 0.1 eq) was added. The Suspension was stirred for 1 h at room temperature. The resulting clear solution was neutralized with saturated aqueuous $NaHCO_3$-solution and then concentrated to half volume. The residue was taken up in EtOAc (150 mL) and washed with brine. Subsequently, the organic phase was dried over Na_2SO_4, filtered and the solvent was removed *in vacuo* to deliver the product (78.0 g, 300 mmol, quant.) as colorless solid.

TLC (Isohexane/EtOAC 1:4): R_f = 0.57.

^{1}H-NMR (300 MHz, $CDCl_3$, 27°C): δ = 5.35 (d, $^3J_{H1,C1OH}$ = 2.6 Hz, 1H; C1H), 4.78 (dd, $^3J_{H3,H2}$ = 5.9 Hz, $^3J_{H3,H4}$ = 3.7 Hz, 1H; C3H), 4.58 (d, $^3J_{H2,H3}$ = 5.9 Hz, 1H; C2H), 4.42 – 4.34 (m, 1H; C5H), 4.15 (dd, $^3J_{H4,H5}$ = 7.1 Hz, $^3J_{H4,H3}$ = 3.6 Hz, 1H; C4H), 4.04 (d, $^3J_{H6a,H5}$ = 3.7 Hz, 1H; C6Ha), 4.03 (d, $^3J_{H6b,H5}$ = 2.8 Hz Hz, 1H; C6Hb), 3.58 (d, $^3J_{C1OH,H1}$ = 2.6 Hz, 1H; C1OH), 1.44 (s, 3H; CH_3), 1.43 (s, 3H; CH_3), 1.35 (s, 3H; CH_3), 1.30 (s, 3H; CH_3) ppm.

^{13}C-NMR (75 MHz, $CDCl_3$, 27°C): δ = 112.5 (C1), 109.1 ($C(CH_3)_2$), 101.1 ($C(CH_3)_2$), 85.5 (C2), 80.0 (C4), 79.6 (C3), 73.2 (C5), 66.4 (C6), 26.7 (CH_3), 25.8 (CH_3), 25.1 (CH_3), 24.4 (CH_3) ppm.

HRMS (ESI$^+$): calc. for $[C_{12}H_{20}O_6 + NH_4]^+$: 278.1598, found.: 278.1603.

1-*O*-Acetyl-2,3:5,6-Diisopropylidene-α-D-mannofuranose [7] [11]

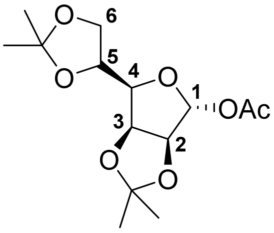

2,3:5,6-Di-*O*-isopropylidene-α-D-mannofuranose (78.0 g, 300 mmol, 1.0 eq.) was dissolved in pyridine (60 mL) and Ac_2O was added at 0 °C (55.0 mL, 582 mmol, 1.9 eq.). The solution was stirred for 1 h at 0 °C and then at room temperature overnight. Subsequently the mixture was cooled to 0 °C again and EtOH (150 mL)was added. After removing the solvent in vacuo, the residue was taken up in EtOAc (200 mL) and washed with HCl (1 M, 200 mL) and saturated aqueous $NaHCO_3$-solution (100 mL). After drying the organic phase over Na_2SO_4 and filtration, the solvent was removed *in vacuo* to give the product as colorless oil.

TLC [Isohexane/EtOAC (1:4)]: R_f = 0.65.

^{1}H-NMR (600 MHz, $CDCl_3$): δ = 6.12 (s, 1H, C1H), 4.85 (dd, $^3J_{C3H\text{-}C2H}$ = 5.9 Hz, $^3J_{C3H\text{-}C4H}$ = 3.6 Hz, 1H, C3H), 4.70 (d, $^3J_{C2H\text{-}C3H}$ = 5.9 Hz, 1H, C2H), 4.40 (ddd, $^3J_{C5H\text{-}C4H}$ = 8.0 Hz, $^3J_{C5H\text{-}C6Ha}$ = 6.1 Hz, $^3J_{C5H\text{-}C6Hb}$ = 4.2 Hz, 1H, C5H), 4.12 (dd, $^3J_{C6Ha\text{-}C6Hb}$ = 8.8 Hz, $^3J_{C6Ha\text{-}C5H}$ = 6.1 Hz, 1H, C6Ha), 4.07 (dd, $^3J_{C6Hb\text{-}C6Ha}$ = 8.8 Hz, $^3J_{C6Hb\text{-}C5H}$ = 4.2 Hz, 1H, C6Hb), 4.06 (dd, $^3J_{C4H\text{-}C5H}$ = 8.0 Hz, $^3J_{C4H\text{-}C3H}$ = 3.6 Hz, 1H, C4H), 2.07 (s, 3H, O_2CCH_3) 1.49 (s, 3H, $C(CH_3)_2$), 1.46 (s, 3H, $C(CH_3)_2$), 1.38 (s, 3H, $C(CH_3)_2$), 1.34 (s, 3H, $C(CH_3)_2$) ppm.

^{13}C-NMR (150 MHz, $CDCl_3$): δ = 169.59 (O_2CCH_3), 113.76 ($C(CH_3)_2$), 109.84 ($C(CH_3)_2$), 109.52 (C1), 100.96 (C2), 82.70 (C4), 79.46 (C3), 73.04 (C5), 67.30 (C6), 27.16 ($C(CH_3)_2$), 26.10 ($C(CH_3)_2$), 25.29 ($C(CH_3)_2$), 24.83 ($C(CH_3)_2$), 21.24 (O_2CCH_3) ppm.

HRMS (ESI+) m/z: calc. for $[C_{14}H_{22}O_7 + NH_4]^+$: 320.1704, found: 320.1706.

1-*O*-Acetyl-2,3-isopropylidene-α-D-mannofuranose [7] [22]

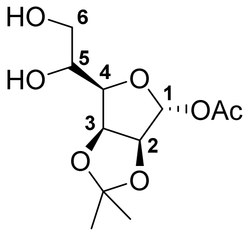

1-*O*-Acetyl-2,3:5,6-Diisopropylidene-α-D-mannofuranose was dissolved in aqueous acetic acid (64%, 500 mL) and stirred for 4 h at 55 °C. The solvent was then removed in vacuo to give the product as yellow oil.

TLC [Isohexane/EtOAC (1:4)]: R_f = 0.16.

^{1}H-NMR (600 MHz, $CDCl_3$, 27 °C): δ = 6.15 (s, 1H; C1H), 4.91 (dd, $^3J_{H3,H2}$ = 5.9 Hz, $^3J_{H3,H4}$ = 3.8 Hz, 1H; C3H), 4.69 (d, $^3J_{H2,H3}$ = 5.9 Hz, 1H; C2H), 4.09 (dd, $^3J_{H4,H5}$ = 8.2 Hz, $^3J_{H4,H3}$ = 3.8 Hz, 1H; C4H), 4.02 – 3.99 (m, 1H; C5H), 3.85 (dd, $^2J_{H6a,H6b}$ = 11.5 Hz, $^3J_{H6a,H5}$ = 3.3 Hz, 1H; C6Ha), 3.71 (dd, $^2J_{H6b,H6a}$ = 11.5 Hz, $^3J_{H6b,H5}$ = 5.5 Hz, 1H; C6Hb), 2.06 (s, 3H; O_2CCH_3), 1.49 (s, 3H; $C(CH_3)_2$), 1.34 (s, 3H; $C(CH_3)_2$) ppm.

^{13}C-NMR (150 MHz, $CDCl_3$, 27 °C): δ = 169.4 (O_2CCH_3), 113.3 ($C(CH_3)_2$), 100.6 (C1), 84.8 (C2), 81.2 (C4), 79.8 (C3), 70.1 (C5), 64.1 (C6), 25.9 ($C(CH_3)_2$), 24.7 ($C(CH_3)_2$), 21.0(O_2CCH_3) ppm.

HRMS (ESI+) m/z: calc. for $[C_{11}H_{18}O_7 + NH_4]^+$: 280.1391, found: 280.1391.

1-*O*-Acetyl-5,6-*O*-methoxymethylen-2,3-*O*-isopropylidene-α-D-mannofuranose [7] [23]

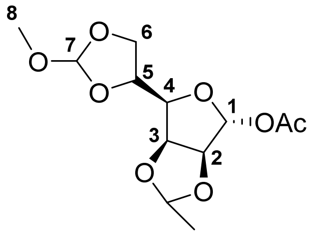

1-*O*-Acetyl-2,3-isopropylidene-α-D-mannofuranose was dissolved in trimethylorthoformiate (165 mL) and stirred at 100 °C for 30 min. After cooling to room temperature and removing the solvent *in vacuo*, the two diastereomeric products were obtained as viscous colorless oil.

TLC [Isohexane/EtOAC (1:4)]: R_f = 0.66.

Isomer A:

^{1}H-NMR (600 MHz, $CDCl_3$, 27 °C): *δ* = 6.12 (s, 1H; C7H), 5.74 (s, 1H; C1H), 4.87 (dd, $^3J_{H3,H2}$ = 5.9 Hz, $^3J_{H3,H4}$ = 3.7 Hz, 1H; C3H), 4.69 (d, $^3J_{H2,H3}$ = 5.9 Hz, 1H; C2H), 4.42 (dt, $^3J_{H5,H4}$ = 8.2 Hz, $^3J_{H5,H6a}$ = $^3J_{H5,H6b}$ = 6.6 Hz, 1H; C5H), 4.17 (dd, $^3J_{H4,H5}$ = 8.3 Hz, $^3J_{H4,H3}$ = 3.5 Hz, 1H; C4H), 4.16 – 4.12 (m, 1H; C6Ha), 4.06 – 4.02 (m, 1H; C6Hb), 3.34 (s, 3H; C8H), 2.05 (s, 3H; O_2CCH_3), 1.47 (s, 3H; $C(CH_3)_2$), 1.33 (s, 3H; $C(CH_3)_2$) ppm.

^{13}C-NMR (150 MHz, $CDCl_3$, 27 °C): δ = 169.3 (O_2CCH_3), 116.4 (C1), 113.2 ($C(CH_3)_2$), 100.8 (C7), 84.9 (C2), 82.6 (C4), 79.5 (C3), 72.8 (C5), 67.1 (C6), 52.1 (C8), 25.9 ($C(CH_3)_2$), 24.6 ($C(CH_3)_2$), 21.0 (O_2CCH_3) ppm.

Isomer B:

^{1}H-NMR (600 MHz, $CDCl_3$, 27 °C): δ = 6.13 (s, 1H; C7H), 5.76 (s, 1H; C1H), 4.82 (dd, $^3J_{H3,H2}$ = 5.9 Hz, $^3J_{H3,H4}$ = 3.7 Hz, 1H; C3H), 4.68 (d, $^3J_{H2,H3}$ = 5.9 Hz, 1H; C2H), 4.58 (td, $^3J_{H5,H6a}$ = $^3J_{H5,H6b}$ = 6.6 Hz, $^3J_{H5,H4}$ = 4.9 Hz, 1H; C5H), 4.16 – 4.12 (m, 2H; C4H, C6Ha), 4.06 – 4.02 (m, 1H; C6Hb), 3.31 (s, 3H; C8H), 2.05 (s, 3H; O_2CCH_3), 1.45 (s, 3H; $C(CH_3)_2$), 1.30 (s, 3H;$C(CH_3)_2$) ppm.

^{13}C-NMR (150 MHz, $CDCl_3$, 27 °C): δ = 169.3 (O_2CCH_3), 115.5 (C1), 113.3 ($C(CH_3)_2$), 100.6(C7), 84.9 (C2), 81.4 (C4), 79.0 (C3), 72.7 (C5), 65.6 (C6), 51.7 (C8), 25.7($C(CH_3)_2$), 24.4 ($C(CH_3)_2$), 21.0 (O_2CCH_3) ppm.

HRMS (ESI+) m/z: calc. for $[C_{13}H_{20}O_8 + NH_4]^+$: 322.1496, found: 322.1499.

(2*S*,3*S*,4*R*)-1-*O*-Acetyl-2,3-*O*-isopropylidene-4-vinyl-α-D-erythrofuranose [7] [12]

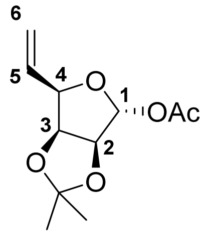

1-*O*-Acetyl-5,6-*O*-methoxymethylen-2,3-*O*-isopropylidene-α-D-mannofuranose was dissolved in Ac_2O (210 mL) and stirred at 130 °C for 5 h. Subsequently the reaction was cooled to 0 °C and EtOH (100 mL) was added. The mixture was stirred at 0 °C for 5 min and then for 15 min at room temperature. After removing the solvent *in vacuo*, the residue was taken up in EtOAc (200 mL) and washed with saturated aqueous $NaHCO_3$-solution. The organic phase was dried over Na_2SO_4, filtered and the solvent was removed *in vacuo* to give the product as brown oil.

TLC [Isohexane/EtOAc (3:1)]: R_f = 0.44.

^{1}H-NMR (600 MHz, $CDCl_3$, 27 °C): δ = 6.18 (bs, 1H; C1H), 5.97 (ddd, $^3J_{H5,H6a}$ = 17.5, $^3J_{H5,H6b}$ = 10.5 Hz, $^3J_{H5,H4}$ = 7.3 Hz, 1H; C5H), 5.43 (dt, $^3J_{H6a,H5}$ = 17.4 Hz, $^2J_{H6a,H6b}$ = $^4J_{H6a,H4}$ = 1.3 Hz, 1H; C6Ha), 5.35 (ddd, $^3J_{H6b,H5}$ = 10.5 Hz, $^2J_{H6b,H6a}$ = 1.4 Hz, $^4J_{H6b,H4}$ = 0.9 Hz, 1H; C6Hb), 4.76 (dd, $^3J_{H3,H2}$ = 5.8 Hz, $^3J_{H3,H4}$ = 3.7 Hz, 1H; C3H), 4.71 (d, $^3J_{H2,H3}$ = 5.8 Hz, 1H; C2H), 4.53 (dd, $^3J_{H4,H5}$ = 7.3 Hz, $^3J_{H4,H3}$ = 3.7 Hz, 1H; C4H), 2.07 (s, 3H; O_2CCH_3), 1.49 (s, 3H; $C(CH_3)_2$), 1.33 (s, 3H; $C(CH_3)_2$) ppm.

^{13}C-NMR (150 MHz, $CDCl_3$, 27°C): δ = 169.5 (O_2CCH_3), 131.5 (C5), 119.7 (C6), 113.2 ($C(CH_3)_2$), 100.6 (C1), 85.3 (C2), 83.3 (C4), 81.2 (C3), 26.1 ($C(CH_3)_2$), 25.0 ($C(CH_3)_2$), 21.1 (O_2CCH_3) ppm.

HRMS (ESI+) m/z: calc. for $[C_{11}H_{16}O_5 + NH_4]^+$: 246.1336, found: 246.1337.

(2*S*,3*S*,4*R*)-2,3-*O*-Isopropylidene-4-vinylerythrofuranose [7] [24]

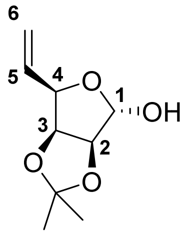

(2*S*,3*S*,4*R*)-1-*O*-Acetyl-2,3-*O*-isopropylidene-4-vinyl-α-D-erythrofuranose was dissolved in MeOH (250 mL) and potassium *tert*-butanolate (6.01 g, 53.6 mmol) was added at 0 °C. The mixture was stirred for 20 min at room temperature and then neutralized with HCl (2 M). The solution was then concentrated *in vacuo* and taken up in DCM and water. The aqueous phase was re-extracted with DCM (3 x 100 mL) and the combined organic phases were dried over Na_2SO_4, filtered and the solvent was removed *in vacuo*. The crude product was purified by column chromatography (Isohexane/EtOAc 6:1) to give the product (23.0 g, 124 mmol, 41% over 5 steps) as colorless solid.

TLC [Isohexane/EtOAC (4:1)]: R_f = 0.20.

^{1}H-NMR (600 MHz, $CDCl_3$, 27 °C): δ = 5.99 (ddd, $^3J_{H5,H6b}$ = 17.5 Hz, $^3J_{H5,H6a}$ = 10.4 Hz, $^3J_{H5,H4}$ = 7.5 Hz, 1H; C5H), 5.41 (d, $^3J_{H1,H2}$ = 2.4 Hz, 1H; C1H), 5.43 – 5.39 (m, 1H; C6Ha), 5.34 (ddd, $^3J_{H6b,H5}$ = 10.4 Hz, $^2J_{H6b,H6a}$ = 1.6 Hz, $^4J_{H6b,H4}$ = 0.8 Hz, 1H; C6Hb), 4.73 (dd, $^3J_{H3,H2}$ = 5.8 Hz, $^3J_{H3,H4}$ = 3.7 Hz, 1H; C3H), 4.64 (d, $^3J_{H2,H3}$ = 5.8 Hz, 1H; C2H), 4.61 (ddd, $^3J_{H4,H5}$ = 7.4 Hz, $^3J_{H4,H3}$ = 3.7 Hz, $^4J_{H4,H6b}$ = 0.7 Hz, 1H; C4H), 2.70 (d, $^3J_{C1\text{-}OH,H1}$ = 2.2 Hz, 1H; C1-OH), 1.47 (s, 3H; $C(CH_3)_2$), 1.32 (s, 3H; $C(CH_3)_2$) ppm.

^{13}C-NMR (150 MHz, $CDCl_3$, 27 °C): δ = 132.2 (C5), 119.2 (C6), 112.6 ($C(CH_3)_2$), 101.1 (C1), 85.8 (C2), 81.5 (2C; C4, C3), 26.0 ($C(CH_3)_2$), 24.8 ($C(CH_3)_2$) ppm.

HRMS (EI) m/z: calc. for [$C_9H_{14}O_4$]: 186.0892, found.: 186.0869.

(3*R*,4*S*,5*R*)-4,5-*O*-Isopropylidenehepta-1,6-dien-3,4,5-triol [7] [13]

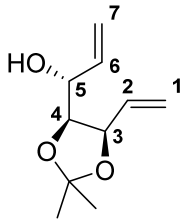

Sodium hydride (14.9 g, 371 mmol, 2.00 eq., 60% dispersed on mineral oil) was suspended in THF (900 mL) and cooled to 0 °C. Subsequently DMSO (46.0 mL, 647 mmol, 3.00 eq.) was added dropwise and the mixture was stirred for 5 min at 0 °C and then for 1 h at room temperature. Methyl triphenylphosphonium bromide (155 g, 435 mmol, 2.00 eq.) was added at 0 °C and the suspension was stirred for 2 h at room temperature. A solution of (2*S*,3*S*,4*R*)-2,3-*O*-isopropylidene-4-vinyl-α-D-erythrofuranose (40. 3 g, 217 mmol, 1.00 eq) in THF (100 mL) was added at 0 °C. Subsequently the reaction mixture was stirred at 65 °C for 3 h. After cooling to room temperature the suspension was diluted with hexane and filtered over celite. The filter residue was washed with hexane (500 mL) and THF (300 mL) and the filtrate was concentrated *in vacuo*. The residue was taken up in water and *tert*-butyl methyl ether and the aqueous phase was extracted with *tert*-butyl methyl ether (3 x 150 mL). The combined organic phases were washed with saturated aqueous $NaHCO_3$-solution and brine. After drying over Na_2SO_4 and filtration the solvent was removed *in vacuo*. Column chromatographic purification (Isohexane/EtOAc 6:1 → 4:1) gave the product (28.4 g, 154 mmol, 71%) as light yellow oil.

TLC [Isohexane/EtOAc (6:1)]: R_f = 0.22.

^{1}H-NMR (600 MHz, $CDCl_3$, 27 °C): δ = 6.00 (ddd, $^3J_{H6,H7a}$ = 17.2 Hz, $^3J_{H6,H7b}$ = 10.3 Hz, $^3J_{H6,H5}$ = 7.9 Hz, 1H; C6H), 5.84 (ddd, $^3J_{H2,H1a}$ = 17.2 Hz, $^3J_{H2,H1b}$ = 10.5 Hz, $^3J_{H2,H3}$ = 5.4 Hz, 1H; C2H), 5.37 (dt, $^3J_{H1a,H2}$ = 17.2 Hz, $^2J_{H1a,H1b}$ = $^4J_{H1a,H3}$ = 1.5 Hz, 1H; C1Ha), 5.35 (ddd, $^3J_{H7a,H6}$ = 17.2 Hz, $^2J_{H7a,H7b}$ = 1.5 Hz, $^4J_{H7a,H5}$ = 1.1 Hz, 1H; C7Ha), 5.28 (ddd, $^3J_{H7b,H6}$ = 10.3 Hz, $^4J_{H7b,H7a}$ = 1.5 Hz, $^4J_{H7b,H5}$ = 0.9 Hz, 1H; C7Hb), 5.22 (dt, $^3J_{H1b,H2}$ = 10.6 Hz, $^2J_{H1b,H1a}$ = $^4J_{H1b,H3}$ = 1.5 Hz, 1H; C1Hb), 4.59 (ddt, $^3J_{H5,H6}$ = 7.7 Hz, $^3J_{H5,H4}$ = 6.7 Hz, $^4J_{H5,H7a}$ = $^4J_{H5,H7b}$ = 0.9 Hz, 1H; C5H), 4.13 – 4.09 (m, 1H; C3H), 4.07 (dd, $^3J_{H4,H5}$ = 6.5 Hz, $^3J_{H4,H3}$ = 5.5 Hz, 1H; C4H), 2.37 (d, $^3J_{C3\text{-}OH,H3}$ = 5.2 Hz, 1H; C3-OH), 1.52 (s, 3H; $C(CH_3)_2$), 1.38 (s, 3H; $C(CH_3)_2$) ppm.

^{13}C-NMR (150 MHz, $CDCl_3$, 27 °C): δ = 136.7 (C2), 133.9 (C6), 119.4 (C7), 117.0 (C1), 108.8 ($C(CH_3)_2$), 80.6 (C4), 78.9 (C5), 70.5 (C3), 27.4 ($C(CH_3)_2$), 25.0 ($C(CH_3)_2$) ppm.

HRMS (ESI+) m/z: calc. for $[C_{10}H_{16}O_3 + NH_4]^+$: 202.1438 found: 202.1439.

(3*R*,4*S*,5*R*)-4,5-*O*-Isopropylidenecyclopent-1-en-3,4,5-triol [7] [14]

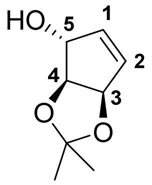

(3*R*,4*S*,5*R*)-4,5-*O*-Isopropylidenehepta-1,6-dien-3,4,5-triol (5.00 g, 27.1 mmol, 1.00 eq.) was dissolved in DCM (85 mL) and Grubbs-I-catalyst (115 mg, 0.140 mmol, 0.005 eq.) was added. The solution was stirred at room temperature for 26 h and the solvent was removed *in vacuo*. Column chromatographic purification (Isohexane/EtOAc 3:1 → 2:1) gave the product (4.12 g, 26.3 mmol, 97%) as colorless oil.

TLC [Isohexane/EtOAc (3:1)]: R_f = 0.10.

^{1}H-NMR (600 MHz, $CDCl_3$, 27 °C): δ = 6.01 – 5.99 (m, 1H; C2H), 5.89 – 5.86 (m, 1H; C1H), 5.27 (dtd, $^3J_{H3,H4}$ = 5.7 Hz, $^3J_{H3,H2}$ = $^4J_{H3,H1}$ = 1.7 Hz, $^5J_{H3,H5}$ = 1.0 Hz, 1H; C3H), 4.76 (bs, 1H; C5H), 4.49 (d, $^3J_{H4,H5}$ = 5.7 Hz, 1H; C4H), 2.58 (bs, 1H; C5-OH), 1.38 (s, 3H; $C(CH_3)_2$), 1.33 (s, 3H; $C(CH_3)_2$) ppm.

^{13}C-NMR (150 MHz, $CDCl_3$, 27 °C): δ = 135.4 (C2), 134.7 (C1), 111.7 ($C(CH_3)_2$), 85.9 (C4),84.2 (C3), 80.9 (C5), 27.3 ($C(CH_3)_2$), 25.7 ($C(CH_3)_2$) ppm.

HRMS (ESI−) *m/z*: calc. for $[C_8H_{11}O_{13} + HCO_2]^-$: 201.0768, found: 201.0771.

(3*R*,4*S*,5*R*)-3-*O*-Trichloacetimidoyl-4,5-*O*-isopropylidenecyclopent-1-en-3,4,5-triol [8] [25]

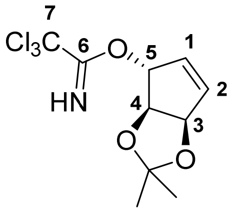

(3*R*,4*S*,5*R*)-4,5-*O*-Isopropylidenecyclopent-1-en-3,4,5-triol (3.50 g, 19.2 mmol, 1.00 eq.) was dissolved in DCM (60 mL) and DBU (4.73 mL, 25.0 mmol, 1.30 eq.) and trichloroacetonitrile (2.89 mL, 28.8 mmol, 1.50 eq.) were added at 0 °C. The solution was stirred for 5 min at 0 °C and then for 15 min at room temperature. The reaction was stopped by adding saturated aqueuous $NaHCO_3$-solution. The aqueous phase was extracted with DCM (4 x 150 mL) and the combined organic phases were dried over Na_2SO_4, filtered and the solvent was removed *in vacuo*. Column chromatographic purification (Isohexane/EtOAc 10:1) gave the product (4.67 g, 15.4 mmol, 80%) as colorless solid.

TLC [Isohexane/EtOAc (10:1)]: R_f = 0.38.

^{1}H-NMR (600 MHz, $CDCl_3$, 27 °C): δ = 8.47 (bs, 1H; NH), 6.22 – 6.19 (m, 1H; C2H), 6.03 (dtd, $^3J_{H1,H2}$ = 5.9 Hz, $^3J_{H1,H5}$ = $^4J_{H1,H3}$ = 1.8 Hz, $^4J_{H1,H4}$ = 0.8 Hz, 1H; C1H), 5.76 (bs, 1H; C5H), 5.32 (ddd, $^3J_{H3,H4}$ = 5.8 Hz, $^3J_{H3,H1}$ = 2.6 Hz, $^4J_{H3,H2}$ = 1.7 Hz, 1H; C3H), 4.69 (d, $^3J_{H4,H5}$ = 5.8 Hz, 1H; C4H), 1.44 (s, 3H; $C(CH_3)_2$), 1.37 (s, 3H; $C(CH_3)_2$) ppm.

^{13}C-NMR (150 MHz, $CDCl_3$, 27 °C): δ = 162.0 (C6), 138.4 (C2), 130.7 (C1), 112.4 ($C(CH_3)_2$), 91.1 (C7), 87.6 (C5), 84.0 (C3), 82.8 (C4), 27.3 ($C(CH_3)_2$), 25.7 ($C(CH_3)_2$) ppm.

HRMS (ESI+) m/z: calc. for $[C_{10}H_{12}NO_3Cl_3 + H]^+$: 299.9956, found: 299.9959.

(3*S*,4*R*,5*S*)-3-Trichloracetylamino-4,5-*O*-isopropylidenecyclopent-1-en-4,5-diol [8] [15]

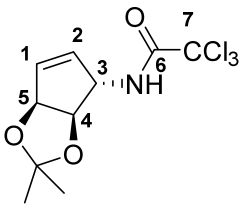

(3*R*,4*S*,5*R*)-3-*O*-Trichloacetimidoyl-4,5-*O*-isopropylidenecyclopent-1-en-3,4,5-triol (4.63 g, 15.4 mmol) was dissolved in *o*-xylene (140 mL). The solution was degassed by freeze-pump-thaw (3x) and was then stirred for 3.5 h at 150 °C. After removing the solvent *in vacuo* and column chromatographic purification (Isohexane/EtOAc 1:7 → 1:3) the product (4.34 g, 14.4 mmol, 94%) was received as a colorless solid.

TLC [Isohexane/EtOAc (7:1)]: R_f = 0.15.

^{1}H-NMR (300 MHz, $CDCl_3$, 27 °C): δ = 6.68 – 6.57 (m, 1H; NH), 6.13 (dtd, $^3J_{H1,H2}$ = 5.7 Hz, $^4J_{H1,H3}$ = $^3J_{H1,H5}$ = 1.1 Hz, $^4J_{H1,H4}$ = 0.5 Hz, 1H; C1H), 5.82 (ddt, $^3J_{H2,H1}$ = 5.7 Hz, $^3J_{H2,H3}$ = 2.5 Hz, $^4J_{H2,H4}$ = $^4J_{H2,H5}$ = 0.9 Hz, 1H; C2H), 5.32 (dtd, $^3J_{H5,H4}$ = 5.7 Hz, $^3J_{H5,H1}$ = $^4J_{H5,H2}$ = 1.7 Hz, $^5J_{H5,H3}$ = 1.0 Hz, 1H; C5H), 4.83 (ddtd, $^3J_{H3,NH}$ = 7.6 Hz, $^3J_{H3,H2}$ = 2.5 Hz, $^3J_{H3,H4}$ = $^4J_{H3,H1}$ = 1.6 Hz, $^5J_{H5,H3}$ = 0.9 Hz, 1H; C3H), 4.58 – 4.54 (m, 1H; C4H), 1.43 (s, 3H; $C(CH_3)_2$), 1.35 (s, 3H; $C(CH_3)_2$) ppm.

^{13}C-NMR (75 MHz, $CDCl_3$, 27 °C): δ = 161.5 (C6), 137.1 (C1), 130.5 (C2), 112.0 ($C(CH_3)_2$), 92.2 (C6), 84.4 (C5), 83.7 (C4), 63.1 (C3), 27.3 ($C(CH_3)_2$), 25.7 ($C(CH_3)_2$) ppm.

HRMS (ESI+) m/z: calc. for $[C_{10}H_{12}NO_3Cl_3 + NH_4]^+$: 317.0216, found.: 317.0224.

(3*S*,4*R*,5*S*)-(*N*-(9*H*-Fluoren-9-yl)methoxycarbonyl)-4,5-*O*-isopropylidenecyclopent-1-en-4,5-diol [26]

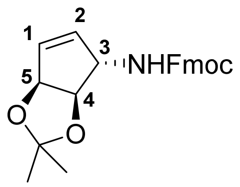

To a solution of (3*S*,4*R*,5*S*)-3-Trichloracetylamino-4,5-*O*-isopropylidenecyclopent-1-en-4,5-diol (6.03 g, 20.1 mmol, 1.0 eq.) in methanol (200 mL) aqueous NaOH (2M, 135 mL) was added and the solution was stirred at room temperature for 14 h. Subsequently the solution was neutralized with HCl (2M) and the solvent was removed *in vacuo*. The residue was taken up in 1,4-dioxane (150 mL), saturated aqueous $NaHCO_3$-solution (30 mL) and water (20 mL) and Fmoc-OSu (8.88 g, 26.3 mmol, 1.30 eq.) was added at 0 °C. The suspension was stirred for 30 min at 0 °C and then for 2.5 h at room temperature. The suspension was then diluted with water (200 mL) and the aqueous phase was extracetd with EtOAc (3 x 150 mL). After drying the combined organic phases over Na_2SO_4 and filtration the solvent was removed *in vacuo*. Column chromatographic purification (Isohexane/EtOAc 3:1) gave the product (6.04 g, 16.0 mmol, 80%) as colorless solid.

TLC [Isohexane/EtOAc (3:1)]: R_f = 0.17.

^{1}H-NMR (300 MHz, $CDCl_3$, 27 °C): δ = 7.79 – 7.74 (m, 2H; C4″H), 7.64 – 7.55 (m, 2H;C1″H), 7.40 (t, $^3J_{H3'',H2''}$ = $^3J_{H3'',H4''}$ = 7.2 Hz, 2H; C3″H), 7.32 (td, $^3J_{H2'',H1''}$ = $^3J_{H2'',H3''}$ = 7.4 Hz, $^4J_{H2'',H4''}$ = 1.2 Hz, 2H; C2″H), 6.02 – 5.97 (m, 1H; C1′H), 5.79 – 5.73 (m, 1H; C2′H), 5.26 –5.19 (m, 1H; C5′H), 4.83 – 4.74 (m, 1H; NH), 4.69 – 4.63 (m, 1H; C3′H), 4.50 – 4.43 (m, 3H; OCH_2CH, C4′H), 4.26 – 4.18 (m, 1H; OCH_2CH), 1.43 (s, 3H; CH_3), 1.35 (s, 3H; CH_3) ppm.

^{13}C-NMR (75 MHz, $CDCl_3$, 27 °C): δ = 155.6 (NHCO), 143.8 (2C; C4″b), 141.3 (2C; C4″a),135.5 (C1′), 132.0 (C2′), 127.7 (2C; C3″), 127.0 (2C; C2″), 124.9 (2C; C1″), 120.0 (C4″),119.9 (C4″), 111.6 ($C(CH_3)_2$), 84.7 (C4′), 84.3 (C5′), 66.6 (OCH2CH), 62.7 (C3′), 47.2(OCH_2CH), 27.3 (CH_3), 25.7 (CH_3) ppm.

HRMS (ESI+) m/z: calc. for $[C_{23}H_{23}O_4N + Na]^+$: 400.1519, found: 400.1519.

(3*S*,4*R*,5*S*)-((*N*-(9*H*-Fluoren-9-yl)methoxycarbonyl))-cyclopent-1-ene-4,5-diol [27]

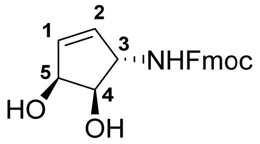

(3*S*,4*R*,5*S*)-(*N*-(9*H*-Fluoren-9-yl)methoxycarbonyl)-4,5-*O*-isopropylidenecyclopent-1-en-4,5-diol (3.56 g, 9.44 mmol) was dissolved in glacial acetic acid (60 mL), water (12 mL) and EtOAc (21 mL). The solution was stirred at 50 °C for 24 h. After removing the solvent *in vacuo*, column chromatographic purification (DCM → DCM/MeOH 10:1) gave the product (2.81 g, 8.33 mmol, 88%) as colorless solid.

TLC [DCM/MeOH (10:1)]: R_f = 0.47.

^{1}H-NMR (400 MHz, DMSO-d^6, 27 °C): δ = 7.89 (d, $^3J_{H4'',H3''}$ = 7.5 Hz, 2H; C4″H), 7.71 (d, $^3J^{H1'',H2''}$ = 7.4 Hz, 2H; C1″H), 7.47 – 7.39 (m, 3H; NH, C3″H), 7.37 – 7.30 (m, 2H; C2″H), 5.87– 5.82 (m, 1H; C1′H), 5.72 (dd, $^3J_{H2',H1'}$ = 6.1 Hz, $^4J_{H2',H5'}$ = 1.4 Hz, 1H; C2′H), 4.65 (d, $^3J_{C5'\text{-}OH,H5}$ = 6.0 Hz, 1H; C5′-OH), 4.60 (d, $^3J_{C4'\text{-}OH,H4'}$ = 6.9 Hz, 1H; C4′-OH), 4.40 – 4.28 (m, 4H; C3′H, C5′H, OCH_2CH), 4.26 – 4.19 (m, 1H; OCH_2CH), 3.77 (dt, $^3J_{H4',C4'\text{-}OH}$ = 6.5 Hz, $^3J_{H4',H5'}$ = $^3J_{H4',H3'}$= 5.6 Hz, 1H; C4′H) ppm.

^{13}C-NMR (100 MHz, DMSO-d$_6$, 27 °C): δ = 156.0 (NHCO), 143.9 (2C; C4″b),140.7 (2C; C4″a), 134.9 (C2′), 133.7 (C1′), 127.6 (2C; C3″), 127.0 (2C; C2″), 125.2 (2C; C1″), 120.1 (2C; C4″), 75.7 (C4′), 72.5 (C5′), 65.3 (OCH_2CH), 61.4 (C3′), 46.7 (OCH_2CH) ppm.

HRMS (ESI+) m/z: calc. for $[C_{20}H_{19}O_4N + Na]^+$: 360.1206, found: 360.1206.

(3*S*,4*R*,5*S*)-(*N*-(9*H*-Fluoren-9-yl)methoxycarbonyl)-5-*O*-*tert*-butyldimethylsilylcyclopent-1-en-4,5-diol [5]

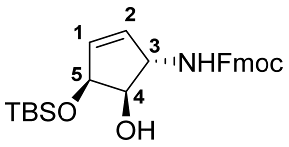

(3*S*,4*R*,5*S*)-((*N*-(9*H*-Fluoren-9-yl)methoxycarbonyl))-cyclopent-1-ene-4,5-diol (2.81 g, 8.34 mmol, 1.0 eq.) was dissolved in DMF (50 mL) and cooled to −55 °C. TBS-OTf (2.30 mL, 9.81 mmol, 1.2 eq.) was added and the solution was stirred at -55 °C for 15 min. Subsequently the solution was poured into saturated aqueuous $NaHCO_3$-solution (500 mL) and the resulting suspension was extracted with DCM (4 x 100 mL). The combined organic phases were dried over Na_2SO_4, filtered and the solvent was removed *in vacuo*. Column chromatographic purification (Isohexane/EtOAc 7:1) gave the product (2.37 g, 5.25 mmol, 63%) as colorless foam.

TLC [Isohexane/EtOAc (1:1)]: R_f = 0.67.

^{1}H-NMR (600 MHz, DMSO-d^6, 27 °C): δ = 7.89 (d, $^{3}J_{H4'',H3''}$ = 7.5 Hz, 2H; C4″H), 7.71 (d, $^{3}J_{H1'',H2''}$ = 7.5 Hz, 2H; C1″H), 7.45 (d, $^{3}J_{NH,H3'}$ = 8.4 Hz, 1H; NH), 7.42 (t, $^{3}J_{H3'',H2''}$ = $^{3}J_{H3'',H4''}$ = 7.4 Hz, 2H; C3″H), 7.33 (t, $^{3}J_{H2'',H1''}$ = $^{3}J_{H2',H3'}$ = 7.4 Hz, 2H; C2″H), 5.85 (dt, $^{3}J_{H1',H2'}$ = 6.0 Hz, $^{3}J_{H1',H5'}$ = $^{4}J_{H1,H3}$ = 2.2 Hz, 1H; C1′H), 5.76 (dd, $^{3}J_{H2',H1'}$ = 6.1 Hz, $^{4}J_{H2',H5'}$ = 1.5 Hz, 1H; C2′H), 4.49 (dd, $^{3}J_{H5',H4'}$ = 5.0 Hz, $^{4}J_{H5',H2'}$ = 2.0 Hz, 1H; C5′H), 4.36 (m, 1H; C3′H), 4.31 (m, 3H; OCH_2CH, C4′-OH), 4.22 (t, $^{3}J_{OCH_2CH,OCH_2CH}$ = 6.9 Hz, 1H; OCH_2CH), 3.75 (m, 1H; C4′H), 0.87 (s, 9H; $SiC(CH_3)_3$), 0.08 (s, 3H; $SiCH_3$), 0.06 (s, 3H; $SiCH_3$) ppm.

^{13}C-NMR (150 MHz, DMSO-d_6, 27 °C): δ = 156.1 (NHCO), 143.9 (C4″b), 143.8 (C4″b), 140.7 (2C; C4″a), 135.9 (C2′), 132.9 (C1′), 127.6 (2C; C3″), 127.0 (2C; C2″), 125.1 (2C; C1″), 120.1 (2C; C4″), 75.8 (C4′), 74.3 (C5′), 65.3 (OCH_2CH), 60.8 (C3′), 46.7 (OCH_2CH), 25.9 (3C; $SiC(CH_3)_3$), 18.1 ($SiC(CH_3)_3$), −4.6 ($SiCH_3$), −4.6 ($SiCH_3$) ppm.

^{29}Si-NMR (80 MHz, $CDCl_3$, 27 °C): δ = 22.49 ppm.

HRMS (ESI+) m/z: calc. for $[C_{26}H_{33}NO_4Si + Na]_+$: 474.2071, found: 474.2070.

Synthesis of precursor 6

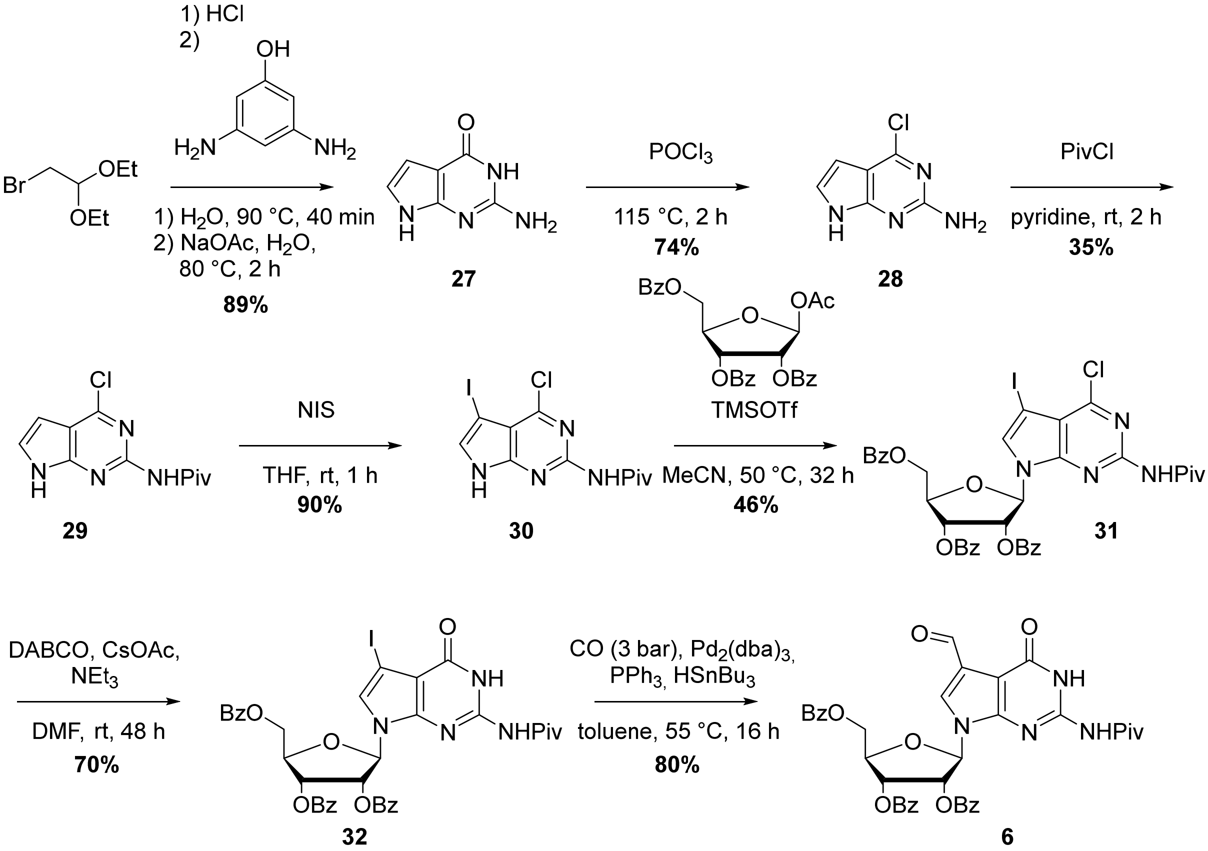

7-Desazaguanine [9] [27]

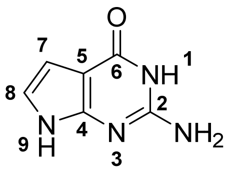

Bromacetaldehyde diethylacetal (64.2 mL, 427 mmol, 1.0 eq.) was suspended in water (210 mL) and concentrated aqueous HCl (9 mL) was added. After stirring for 40 min at 90 °C a colorless solution was obtained to which sodium acetate (40.8 g, 497 mmol, 1.20 eq.) was added after cooling to room temperature. The solution was then added to a suspension of 2,6-Diamino-4-pyrimidinone (60.2 g, 477 mmol, 1.10 eq.) and sodium acetate (21.1 g, 257 mmol, 0.60 eq.) in water (450 mL). The resulting solution was stirred for 2 h at 80 °C. The product precipitated as light red solid, which was filtered off and washed with cold water and acetone. After drying the product at 55 °C on high vacuum the product (56.8 g, 378 mmol, 89%) was obtained as light red solid.

^{1}H NMR (200 MHz, DMSO-d^{6}): δ = 10.81 (s, 1H, NH), 10.11 (s, 1H, NH), 6.45 (dd, $^{3}J_{C7H\text{-}C8H}$ = 3.4 Hz, J_2 = 2.2 Hz, 1H, C7H), 6.03 (dd, $^{3}J_{C7H\text{-}C8H}$ = 3.4 Hz, J_2 = 2.2 Hz, 1H, C8H), 5.90 (s, 2H, NH_2) ppm.

HRMS (EI+) *m/z*: calc. for $[C_6H_6N_4O]^+$: 150.0542, found: 150.0544.

6-Desoxy-6-chloro-7-desazaguanin [9] [28]

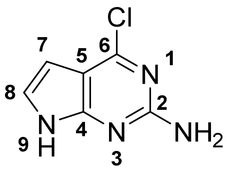

7-Desazaguanine (15.92 g, 106 mmol, 1.0 eq.) was suspended in $POCl_3$ (130 mL) and heated to 115 °C for 2 h. The Suspension was cooled to room temperature and $POCl_3$ was removed *in vacuo*. Ice and ice water were added slowly to the residue and the pH was adjusted to pH = 4 with concentrated aqueuous ammonia. After stirring for 30 min at 0 °C the precipitate was filtered off, washed with ice-cold water and diethyl ether and dried at 55 °C on high vacuum. The product (13.3 g, 78.7 mmol, 74%) was obtained as yellow-beige solid.

^{1}H-NMR (400 MHz, DMSO-d^6, 27 °C): δ = 11.48 (bs, 1H; NH), 7.08 (d, $^3J_{H8,H7}$ = 3.6 Hz, 1H; C8H), 6.47 (bs, 2H; NH_2), 6.24 (d, $^3J_{H7,H8}$ = 3.6 Hz, 1H; C7H) ppm.

^{13}C-NMR (100 MHz, DMSO-d^6, 27 °C): δ = 159.3 (C2), 154.6 (C4), 150.9 (C6), 123.2 (C8), 108.6 (C5), 98.6 (C7) ppm.

HRMS (ESI+) *m/z*: calc. for $[C_6H_5N_4Cl + H]^+$: 169.0276, found: 169.0276.

6-Desoxy-6-chloro-2-pivaloylamino-7-desazaguanine [9] [29]

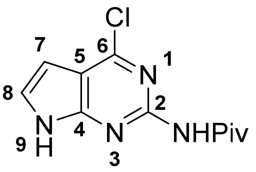

6-Desoxy-6-chloro-7-desazaguanine (11.9 g, 70.3 mmol, 1.0 eq.) was suspended in pyridine (167 mL) and Pivaloylchloride (30.3 mL, 246 mmol, 3.5 eq.) was added dropwise. The reaction mixture was stirred for 2 h at room temperature. Subsequently the solvent was removed *in vacuo* and the residue was taken up in DCM (1 L) and washed with HCl (0.1M, 2 × 500 mL). The combined organic phases were dried over $MgSO_4$, filtered and the solvent was removed *in vacuo*. Column chromatographic purification (DCM/MeOH 98:2) gave the product (6.22 g, 24.6 mmol, 35%) as yellow solid.

TLC [DCM/MeOH (15:1)]: R*f* = 0.45.

1H-NMR (400 MHz, DMSO-d^6, 27 °C): δ = 12.32 (bs, 1H; NH), 10.02 (s, 1H; NHCO), 7.53 (dd, $^3J_{H8,H7}$ = 3.3 Hz, $^3J_{H8,NH}$ = 2.5 Hz, 1H; C8H), 6.52 (dd, $^3J_{H7,H8}$ = 3.5 Hz, $^4J_{H7,NH}$ = 1.8 Hz, 1H; C7H), 1.23 (s, 9H; $C(CH_3)_3$) ppm.

13C-NMR (100 MHz, DMSO-d^6, 27 °C): δ = 175.8 (NHCO), 152.7 (C6), 151.3 (C2), 150.2 (C4), 127.3 (C8), 113.2 (C5), 98.8 (C7), 39.3 ($C(CH_3)_3$), 26.9 (3C; $C(CH_3)_3$) ppm.

HRMS (ESI+) m/z: calc. for $[C_{11}H_{13}N_4ClO + Na]^+$: 275.0670, found: 275.0670.

6-Desoxy-6-chloro-7-iodo-2-pivaloylamino-7-desazaguanine [9] [30]

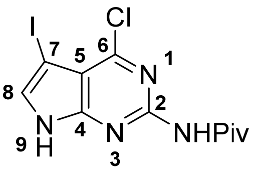

6-Desoxy-6-chloro-2-pivaloylamino-7-desazaguanine (3.68 g, 14.6 mmol, 1.0 eq.) was dissolved in THF (70 mL) and the flask was covered with aluminum foil. *N*-Iodosuccinimide (3.61 g, 16.1 mmol, 1.10 eq.) was added and the solution was stirred for 1 h at room temperature. After removing the solvent *in vacuo* the residue was taken up in DCM (300 mL) and washed with water (2 x 150 mL). The combined organic phases were dried over $MgSO_4$ and the solvent was removed *in vacuo*. Column chromatographic purification (DCM/MeOH 99:1) gave the product (4.98 g, 13.2 mmol, 90%) as yellow solid.

TLC [DCM/MeOH (15:1)]: Rf = 0.56.

^{1}H-NMR (400 MHz, DMSO-d^6, 27 °C): δ = 12.69 (d, $^3J_{NH,H8}$ = 1.7 Hz, 1H; NHCH), 10.09 (s, 1H; NHCO), 7.76 (d, $^3J_{H8,NH}$ = 2.4 Hz, 1H; C8H), 1.22 (s, 9H; $C(CH_3)_3$) ppm.

^{13}C-NMR (100 MHz, DMSO-d^6, 27 °C): δ = 175.8 (NHCO), 152.5 (C6), 151.4 (C2), 150.7 (C4), 132.7 (C8), 112.3 (C5), 51.6 (C7), 39.4 ($C(CH_3)_3$), 26.8 (3C; $C(CH_3)_3$) ppm.

HRMS (ESI+) m/z: calc. for $[C_{11}H_{12}N_4OClI + H]^+$: 378.9817, found: 378.9816.

2′,3′,5′-Tri-*O*-benzoyl-2-*N*-pivaloyl-6-desoxy-6-chloro-7-iodo-7-desazaguanosine [9] [31]

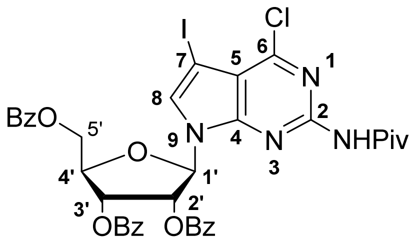

6-Desoxy-6-chloro-7-iodo-2-pivaloylamino-7-desazaguanine (4.45 g, 11.8 mmol, 1.0 eq.) was suspended in acetonitrile (82 mL) and *N*,*O*-Bistrimethylsilylacetamide (3.45 mL, 14.1 mmol, 1.2 eq.) was added. After stirring for 10 min at room temperature TMSOTf (2.77 mL, 15.3 mmol, 1.30 eq) was added and the mixture was stirred at 50 °C. 1-*O*-Acetyl-2,3,5-tri-*O*benzoyl-β-D-ribofuranose (11.9 g, 23.6 mmol, 2.00 eq.) was added over a course of 24 h in three portions and the mixture was left stirring for another 8 h at 50 °C. The solution was diluted with DCM (1 L) and washed with saturated aqeueous $NaHCO_3$-solution and brine. The organic phase was dried over $MgSO_4$, filtered and the solvent was removed in vacuo. Column chromatographic purification (Isohexane/EtOAc 4:1) gave the product (4.46 g, 5.42 mmol, 46%) as yellow foam.

TLC [Isohexane/EtOAc (3:1)]: R*f* = 0.24.

^{1}H-NMR (600 MHz, $CDCl_3$, 27 °C): *δ* = 8.25 (s, 1H; NHCO), 8.01 – 7.94 (m, 6H; ArH), 7.58 – 7.52 (m, 3H; ArH), 7.44 – 7.34 (m, 6H; ArH), 7.38 (s, 1H; C8H), 6.50 (t, $^3J_{H3',H2'}$ = 3$J_{H3',H4'}$= 6.0 Hz, 1H; C3′H), 6.41 (d, $^3J_{H1',H2'}$ = 3.9 Hz, 1H; C1′H), 6.25 (dd, $^3J_{H2',H3'}$ = 5.8 Hz, $^3J_{H2',H1'}$ = 3.9 Hz, 1H; C2′H), 4.88 (dd, $^2J_{H5'a,H5'b}$ = 12.2 Hz, $^3J_{H5'a,H4'}$ = 3.4 Hz, 1H; C5′Ha), 4.82 – 4.79 (m, 1H; C4′H), 4.69 (dd, $^2J_{H5'b,H5'a}$ = 12.2 Hz, $^3J_{H5'b,H4'}$ = 4.4 Hz, 1H; C5′Hb), 1.31 (s, 9H; $C(CH_3)_3$) ppm.

^{13}C-NMR (150 MHz, $CDCl_3$, 27 °C): δ = 175.1 ($COC(CH_3)_3$), 166.1 (ArCO), 165.4 (ArCO), 165.2 (ArCO), 153.3 (C6), 151.5 (C4), 151.4 (C2), 133.6 (Ar), 133.5 (Ar), 133.3 (Ar), 132.0 (C8), 129.9 (2C; Ar), 129.8 (2C; Ar), 129.7 (2C; Ar), 129.5 (2C; Ar), 129.3 (Ar), 128.8 (Ar), 128.6 (2C, Ar), 128.4 (3C; Ar), 114.1 (C5), 88.3 (C1′), 80.0 (C4′), 74.5 (C2′), 71.3 (C3′), 63.4 (C5′), 53.5 (C7), 40.2 ($C(CH_3)_3$), 27.3 (3C; $C(CH_3)_3$) ppm.

HRMS (ESI+) *m/z*: calc. for $[C_{37}H_{32}O_8N_4ClI + H]^+$: 823.1026, found: 823.1028.

2′,3′,5′-Tri-*O*-benzoyl-2-*N*-pivaloyl-7-iodo-7-desazaguanosin [9] [32]

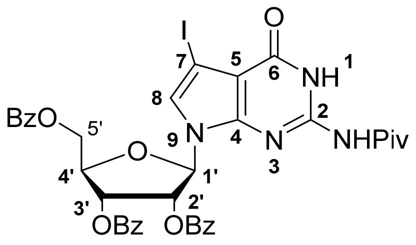

2′,3′,5′-Tri-*O*-benzoyl-2-*N*-pivaloyl-6-desoxy-6-chloro-7-iodo-7-desazaguanosine (4.35 g, 5.28 mmol, 1.00 eq.) was dissolved in DMF (16 mL) and the flask was covered with aluminum foil. CsOAc (3.04 g, 15.9 mmol, 3.00 eq.), 1,4-Diaza[2.2.2]bicyclooctane (600 mg, 5.34 mmol, 1.00 eq.) and triethylamine (2.23 mL, 16.0 mmol, 3.00 eq.) were added and the solution was stirred for 48 h at room temperature. Water (20 mL) was added and stirring continued for another 30 min at room temperature. EtOAc and brine were added and the phases were separated. The aqueous phase was re-extracted with EtOAc (200 mL). The combined organic phases were washed with brine, dried over $MgSO_4$, filtered and the solvent was removed *in vacuo*. Column chromatographic purification (Isohexane/EtOAc 2:1) gave the product (3.00 g, 3.73 mmol, 70%) as colorless foam.

TLC [Isohexane/EtOAc (1:1)]: R*f* = 0.40.

^{1}H-NMR (600 MHz, $CDCl_3$, 27 °C): δ = 11.56 (s, 1H; NHCO), 8.76 (s, 1H; NHCOC(CH_3)$_3$), 8.00 – 7.97 (m, 2H; ArH), 7.92 – 7.89 (m, 2H; ArH), 7.78 – 7.75 (m, 2H; ArH), 7.62 – 7.53 (m, 3H; ArH), 7.46 – 7.42 (m, 2H; ArH), 7.40 – 7.32 (m, 4H; ArH), 6.96 (s, 1H; C8H), 6.85 (dd, $^3J_{H3',H4'}$ = 8.4 Hz, $^3J_{H3',H2'}$ = 5.0 Hz, 1H; C3′H), 6.46 (dd, $^3J_{H2',H3'}$ = 5.0 Hz, $^3J_{H2',H1'}$ = 1.7 Hz, 1H; C2′H), 6.01 (d, $^3J_{H1',H2'}$ = 1.7 Hz, 1H; C1′H), 4.77 (dt, $^3J_{H4',H3'}$ = 8.4 Hz, $^3J_{H4',H5'a}$ = $^3J_{H4',H5'b}$ = 3.0 Hz, 1H; C4′H), 4.72 (dd, $^2J_{H5'a,H5'b}$ = 12.5 Hz, $^3J_{H5'a,H4'}$ = 2.9 Hz, 1H; C5′Ha), 4.64 (dd, $^2J_{H5'b,H5'a}$ = 12.5 Hz, $^3J_{H5'b,H4'}$ = 3.2 Hz, 1H; C5′Hb), 1.35 (s, 9H; C(CH_3)$_3$) ppm.

^{13}C-NMR (150 MHz, $CDCl_3$, 27 °C): δ = 180.1 (COC(CH_3)$_3$), 166.0 (2C; ArCO), 165.1 (ArCO), 156.6 (C6), 146.4 (C2), 146.2 (C4), 133.7 (2C; Ar), 133.5 (Ar), 129.8 (2C; Ar), 129.7 (2C; Ar), 129.2 (2C; Ar), 128.9 (Ar), 128.7 (Ar), 128.6 (3C; Ar), 128.5 (2C; Ar), 128.4 (2C; Ar), 127.1 (C8), 106.5 (C5), 89.3 (C1′), 78.5 (C4′), 74.4 (C2), 70.5 (C3′), 61.4 (C5′), 56.0 (C7), 40.2 (COC(CH_3)$_3$), 26.8 (3C; C(CH_3)$_3$) ppm.

HRMS (ESI+) *m/z*: calc. for $[C_{37}H_{33}O_9N_4I + H]^+$: 805.1365, found: 805.1375.

2′,3′,5′-Tri-*O*-benzoyl-2-*N*-pivaloyl-7-formyl-7-desazaguanosine [9] [6]

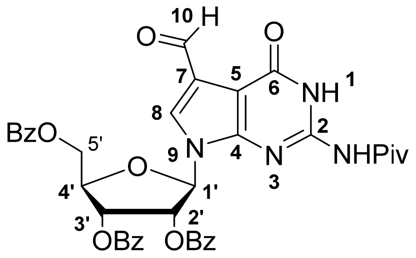

2′,3′,5′-Tri-*O*-benzoyl-2-*N*-pivaloyl-7-iodo-7-desazaguanosine (810 mg, 1.00 mmol, 1.00 eq.), triphenylphosphine (160 mg, 600 µmol, 0.60 eq.) and $Pd_2(dba)_3$ (94.0 mg, 100 µmol, 0.10 eq.) were dissolved in toluene (12.5 mL). The flask was flushed three times with CO gas and a CO-atmosphere of 3.5 bar was maintained in the flask. The solution was stirred at 55 °C and tributyltin hydride (330 µL, 1.20 mmol, 1.2 eq) in toluene (0.33 mL) was added over a course of 16 h with a syringe pump. The crude reaction mixture was filtered through a flash column (EtOAc) and the solvent was removed *in vacuo*. Column chromatographic purification (Isohexane/EtOAc 2:1) gave the product (570 mg, 810 µmol, 80%) as brown foam.

TLC [Isohexane/EtOAc (1:1)]: R*f* = 0.28.

^{1}H-NMR (600 MHz, $CDCl_3$, 27 °C): δ = 11.79 (s, 1H; NHCO), 10.37 (s, 1H; CHO), 8.84 (s, 1H; NHCOC(CH_3)$_3$), 8.00 – 7.97 (m, 2H; ArH), 7.93 – 7.89 (m, 2H; ArH), 7.78 – 7.74 (m, 2H; ArH), 7.65 – 7.52 (m, 3H; ArH), 7.61 (s, 1H; C8H), 7.47 – 7.43 (m, 2H; ArH), 7.41 – 7.35 (m, 2H; ArH), 7.36 – 7.31 (m, 2H; ArH), 6.89 (dd, $^3J_{H3,H4'}$ = 8.4 Hz, $^3J_{H3',H2'}$ = 5.0 Hz, 1H; C3′H), 6.50 (dd, $^3J_{H2',H3'}$ = 5.0 Hz, $^3J_{H2',H1'}$ = 1.7 Hz, 1H; C2′H), 6.07 (d, $^3J_{H1',H2'}$ = 1.7 Hz, 1H; C1′H), 4.82 (dt, $^3J_{H4',H3'}$ = 8.4 Hz, $^3J_{H4',H5'a}$ = $^3J_{H4',H5'b}$ = 3.3 Hz, 1H; C4′H), 4.76 (dd, $^2J_{H5'a,H5'b}$ = 12.5 Hz, $^3J_{H5'a,H4'}$ = 3.2 Hz, 1H; C5′Ha), 4.67 (dd, $^2J_{H5'b,H5'a}$ = 12.5 Hz, $^3J_{H5'b,H4'}$ = 3.4 Hz, 1H; C5′Hb), 1.38 (s, 9H; C(CH_3)$_3$) ppm.

^{13}C-NMR (150 MHz, $CDCl_3$, 27 °C): δ = 186.0 (CHO), 180.3 (COC(CH_3)$_3$), 166.1 (ArCO), 166.0 (ArCO), 165.1 (ArCO), 156.7 (C6), 147.3 (C4), 147.2 (C2), 133.9 (Ar), 133.8 (Ar), 133.6 (Ar), 129.8 (4C; Ar), 129.2 (2C; Ar), 128.9 (Ar), 128.6 (6C; Ar), 128.4 (2C; Ar), 126.6 (C8), 121.0 (C7), 104.7 (C5), 90.4 (C1′), 78.8 (C4′), 74.4 (C2′), 70.6 (C3′), 61.4 (C5′), 40.3 (C(CH_3)$_3$), 26.9 (C(CH_3)$_3$) ppm.

HRMS (ESI+) *m/z*: calc. for $[C_{38}H_{34}O_{10}N_4 + H]^+$: 707.2348, found: 707.2350.

Assembly of GalQ 2 from precursors 4,5 and 6

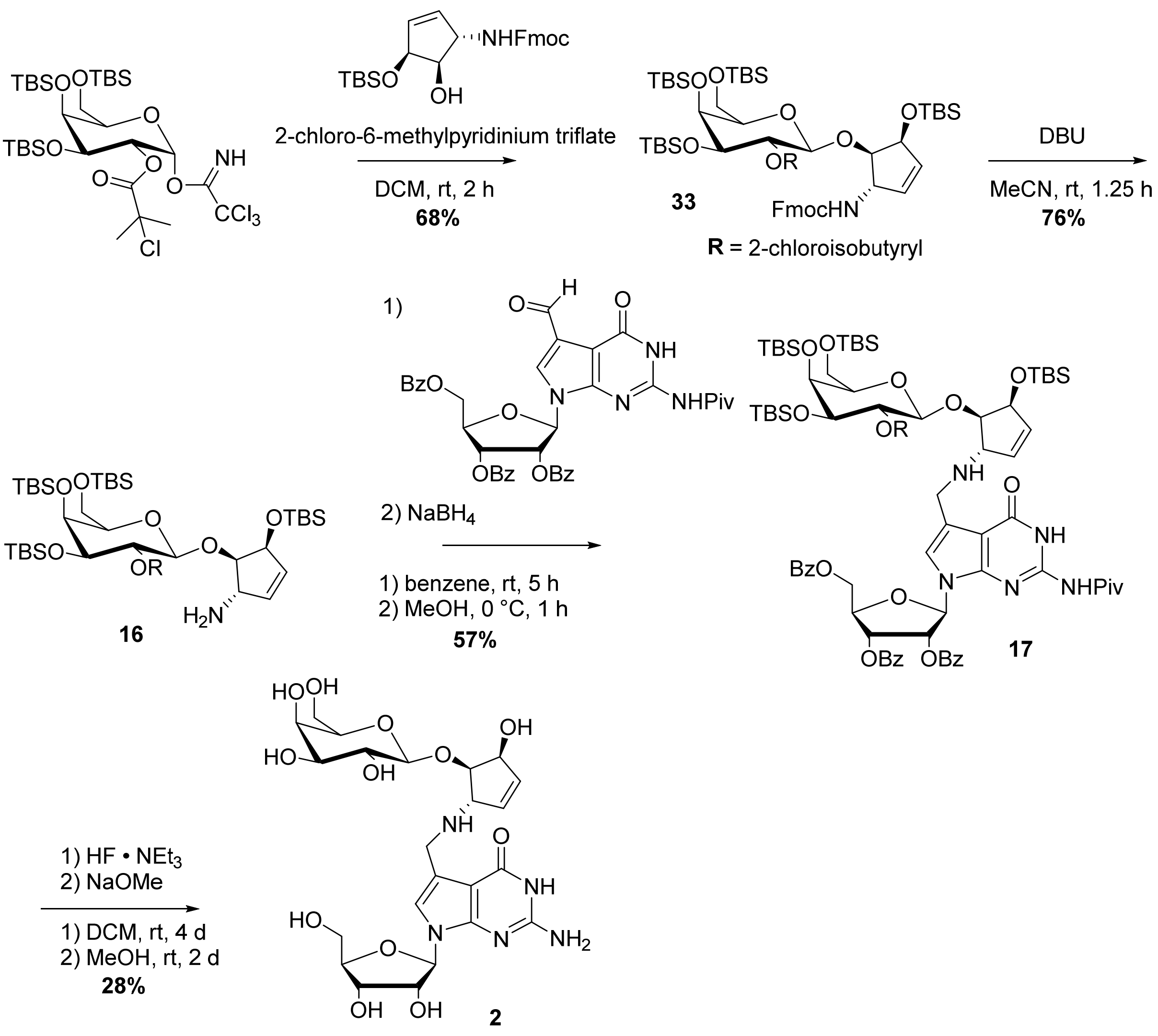

1-*O*-((3*S*,4*R*,5*S*)-((*N*-(9*H*-Fluoren-9-yl)methoxycarbonyl)-5-*O*-(*tert*-butyldimethyl-silyl)-cyclopent-1-en-4-yl))-3,4,6-tri-*O*-*tert*-butyldimethylsilyl-2-*O*-(2-chloroiso-butyryl)-β-D-galactopyranose [33]

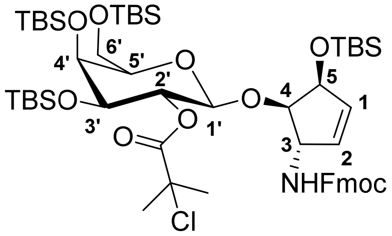

To a solution of 3,4,6-Tri-*O*-*tert*-butyldimethylsilyl-2-*O*-(2-chlorisobutyryl)-D-galactopyranosyl-1-*O*-trichloracetimidate (2.20 g, 2.85 mmol, 1.00 eq.) in *n*-hexane (150 mL) (3*S*,4*R*,5*S*)-(*N*-(9*H*-Fluoren-9-yl)methoxycarbonyl)-5-*O*-*tert*-butyldimethyl-silylcyclopent-1-en-4,5-diol (2.18 g, 4.83 mmol, 1.70 eq.) dissolved in DCM (12 mL) and 3Å molecular sieve was added and the solution was stirred for 2 h at room temperature. Subsequently 2-Chlor-6-methylpyridinium triflate (0.08 g, 0.29 mmol, 0.10 eq.) was added and the solution was stirred for another 2 h at room temperature. The resulting suspension was filtered through a micro filter into saturated aqueous $NaHCO_3$-solution. After separation of the phases, the aqueous phase was extracted with EtOAc (3 x 60 mL). The combined organic phases were dried over $MgSO_4$, filtered and the solvent was removed *in vacuo*. Column chromatographic purification (Isohexane/EtOAc 30:1 → 10:1) gave the product (2.06 g, 1.94 mmol, 68%) as colorless foam.

TLC [Isohexane/EtOAc (8:1)]: R*f* = 0.31.

^{1}H-NMR (400 MHz, $CDCl_3$, 27 °C): δ = 7.79 – 7.75 (m, 2H; C4″H), 7.62 – 7.58 (m, 2H;C1″H), 7.43 – 7.38 (m, 2H; C3″H), 7.35 – 7.29 (m, 2H; C2″H), 5.91 – 5.86 (m, 1H; C1′H),5.80 – 5.75 (m, 1H; C2′H), 5.29 (dd, $^3J_{H2,H3}$ = 9.7, $^3J_{H2,H1}$ = 8.0 Hz, 1H; C2H), 4.86 (d, $^3J_{NH,H3'}$ = 7.4 Hz, 1H; NH), 4.73 (d, $^3J_{H1,H2}$ = 8.0 Hz, 1H; C1H), 4.69 – 4.60 (m, 2H; C3′H, C5′H),4.42 (d, $^3J_{OCH_2CH,OCH_2CH}$ = 6.6 Hz, 2H; OCH_2CH), 4.24 (t, $^3J_{OCH_2CH,OCH_2CH}$ = 6.9 Hz, 1H; OCH_2CH), 4.07 – 4.01 (m, 1H; C4′H), 3.98 (d, $^3J_{H4,H3}$ = 2.1 Hz, 1H; C4H), 3.73 (dd, $^2J_{H6a,H6b}$ = 9.9 Hz, $^3J_{H6a,H5}$ = 7.5 Hz, 1H; C6Ha), 3.71 – 3.68 (m, 1H; C6Hb), 3.66 (dd, $^3J_{H3,H2}$ = 10.0 Hz, $^3J_{H3,H4}$ = 2.3 Hz, 1H; C3H), 3.44 – 3.38 (m, 1H; C5H), 1.77 (s, 6H; $CCl(CH_3)_2$), 0.93 (s, 9H; $SiC(CH_3)_3$), 0.89 (s, 9H; $SiC(CH_3)_3$), 0.88 (s, 9H; $SiC(CH_3)_3$), 0.86 (s, 9H; $SiC(CH_3)_3$), 0.20 (s, 3H; $SiCH_3$), 0.10 (s, 3H; $SiCH_3$), 0.08 (s, 3H; $SiCH_3$), 0.07 (3s, 9H; $SiCH_3$), 0.06 (s, 3H; $SiCH_3$), 0.05 (s, 3H; $SiCH_3$) ppm.

^{13}C-NMR (100 MHz, $CDCl_3$, 27 °C): δ = 170.5 ($COCCl(CH_3)_2$), 155.7 (NHCO), 144.0 (C4″b), 143.8 (C4″b), 141.4 (C4″a), 141.3 (C4″a), 134.7 (C1′), 133.4 (C2′), 127.7 (2C; C3″), 127.0 (2C; C2″), 125.0 (C1″), 124.9 (C1″), 120.0 (2C; C4″), 100.5 (C1), 81.0 (C4′), 75.8 (C5), 74.3 (C2), 74.0 (C3), 73.7 (C5′), 71.4 (C4), 66.6 (OCH_2CH), 65.2 ($CCl(CH_3)_2$), 61.4 (C6), 59.8 (C3′), 47.2 (OCH_2CH), 30.5 ($CCl(CH_3)_3$), 26.2 (2 × 3C; $SiC(CH_3)_3$), 26.0 (3C; $SiC(CH_3)_3$), 25.8 (3C; $SiC(CH_3)_3$), 18.6 ($SiC(CH_3)_3$), 18.4 ($SiC(CH_3)_3$), 18.1 (2C; $SiC(CH_3)_3$), −3.6 ($SiCH_3$), −3.8 ($SiCH_3$), −4.2 ($SiCH_3$), −4.6 ($SiCH_3$), −4.7 ($SiCH_3$), −5.0 ($SiCH_3$), −5.2 ($SiCH_3$), −5.3 ($SiCH_3$) ppm.

HRMS (ESI+) *m/z*: calc. for [$C_{54}H_{90}O_{10}NClSi_4 + NH_4]^+$: 1077.5669, found: 1077.5672.

1-*O*-((3*S*,4*R*,5*S*)-(3-Amino-5-*O*-(*tert*-butyldimethylsilyl)cyclopent-1-en-4-yl))-3,4,6-tri-*Otert*-butyldimethylsilyl-2-*O*-(2-chlorisobutyryl)-β-D-galactopyranose [16]

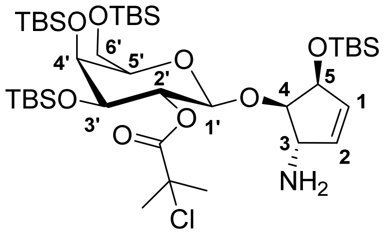

1-*O*-((3*S*,4*R*,5*S*)-((*N*-(9*H*-Fluoren-9-yl)methoxycarbonyl)-5-*O*-(*tert*-butyldimethylsilyl)-cyclopent-1-en-4-yl))-3,4,6-tri-*O*-*tert*-butyldimethylsilyl-2-*O*-(2-chloroisobutyryl)-β-D-galactopyranose (144 mg, 136 µmol, 1.0 eq.) was dissolved in acetonitrile (6 mL) and DBU (0.9 mL, 6.02 mmol) was added. The reaction mixture was stirred for 1.25 h at room temperature. Removal of the solvent *in vacuo* and subsequent column chromatographic purification (Isohexane/EtOAc 5:1 → 4:1) gave the product (87 mg, 104 µmol, 76%) as colorless foam.

TLC [Isohexane/EtOAc (4:1)]: R*f* = 0.05.

^{1}H-NMR (600 MHz, $CDCl_3$, 27 °C): δ = 5.80 (dt, $^3J_{H1',H2'}$ = 6.0 Hz, $^3J_{H1',H5'}$ = $^4J_{H1',H3'}$ = 2.4 Hz, 1H; C1′H), 5.73 (d, $^3J_{H2',H1'}$ = 6.0 Hz, 1H; C2′H), 5.31 (t, $^3J_{H2,H1}$ = $^3J_{H2,H3}$ = 8.3 Hz, 1H; C2H), 4.82 (d, $^3J_{H1,H2}$ = 7.9 Hz, 1H; C1H), 4.51 (ddd, $^3J_{H5',H4'}$ = 5.3 Hz, $^3J_{H5',H1'}$ = 2.7 Hz, $^4J_{H5',H2'}$ = 0.9 Hz, 1H; C5′H), 4.02 – 3.98 (m, 2H; C4H, C3′H), 3.75 – 3.67 (m, 3H; C3H, C6Ha, C6Hb), 3.65 (t, $^3J_{H4',H5'}$ = $^3J_{H4',H3'}$ = 5.6 Hz, 1H; C4′H), 3.40 (t, $^3J_{H5,H6a}$ = $^3J_{H5,H6b}$ = 6.6 Hz, 1H; C5H), 1.80 (s, 3H; $CCl(CH_3)_2$), 1.79 (s, 3H; $CCl(CH_3)_2$), 0.93 (s, 9H; $SiC(CH_3)_3$), 0.90 (s, 9H; $SiC(CH_3)_3$), 0.89 (s, 9H; $SiC(CH_3)_3$), 0.84 (s, 9H; $SiC(CH_3)_3$), 0.20 (s, 3H; $SiCH_3$), 0.13 (s, 3H; $SiCH_3$), 0.11 (s, 3H; $SiCH_3$), 0.10 (s, 3H; $SiCH_3$), 0.06 (4s, 12H; $SiCH_3$) ppm.

^{13}C-NMR (150 MHz, $CDCl_3$, 27 °C): δ = 170.6 (*C*OCCl(CH_3)$_2$), 138.4 (C2′), 132.0 (C1′),101.5 (C1), 86.4 (C4′), 75.9 (C5), 74.9 (C5′), 74.1 (C3), 73.9 (C2), 71.5 (C4), 65.1 (*C*Cl(CH3)2), 61.6 (C6), 59.8 (C3′), 30.8 (CCl(CH_3)$_2$), 30.5 (CCl(CH_3)$_2$), 26.2 (6C; SiC(CH_3)$_3$), 26.0 (3C; SiC(CH_3)$_3$), 25.8 (3C; SiC(CH_3)$_3$), 18.6 (Si*C*(CH_3)$_3$), 18.4 (SiC(CH_3)$_3$), 18.1 (2C; SiC(CH_3)$_3$), −3.6 ($SiCH_3$), −3.9 ($SiCH_3$), −4.2 ($SiCH_3$), −4.6 (2C; $SiCH_3$), −4.9 ($SiCH_3$), −5.2 ($SiCH_3$), −5.3 ($SiCH_3$) ppm.

HRMS (ESI+) *m/z*: calc. for [$C_{39}H_{80}O_8NClSi_4$ + H]+: 838.4722, found: 838.4724.

7-Desaza-7-((((3*S*,4*R*,5*S*)-4-*O*-(3,4,6-tri-*O*-*tert*-butyldimethylsilyl-2-*O*-(2-chlorisobutyryl)-β-D-galactopyranosyl)-5-*O*-(*tert*-butyldimethylsilyl)cyclopent-1-en-3-yl)amino)-methyl)-2-pivaloylamino-2′,3′,5′-tri-*O*-benzoylguanosin [17]

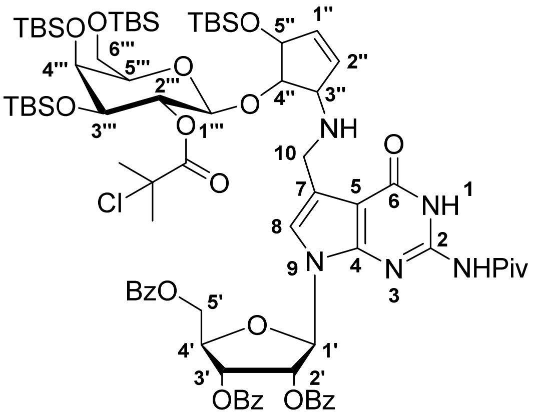

1-*O*-((3*S*,4*R*,5*S*)-(3-Amino-5-*O*-(*tert*-butyldimethylsilyl)cyclopent-1-en-4-yl))-3,4,6-tri-*O*-*tert*-butyldimethylsilyl-2-*O*-(2-chlorisobutyryl)-β-D-galactopyranose (150 mg, 179 µmol, 1.20 eq.) and 2′,3′,5′-Tri-*O*-benzoyl-2-*N*-pivaloyl-7-formyl-7-desazaguanosine (105 mg, 145 µmol, 1.00 eq.) were dissolved in benzene (2 mL) and the solution was stirred at room temperature for 5 h. After removing the solvent *in vacuo* the residue was taken up in methanol (2 mL). The solution was cooled to 0 °C and $NaBH_4$ (20 mg, 0.53 mmol, 3.60 eq) was added. After stirring the solution for 1 h at 0 °C water (0.15 mL) was added. The reaction mixture was stirred for another 10 min at 0 °C before the solvent was removed *in vacuo*. Column chromatographic purification (Isohexane/EtOAc 5:1 → 2:1) gave the product (129 mg, 83 µmol, 57%) as colorless foam.

TLC [Isohexane/EtOAc (1:1)]: R*f* = 0.53.

^{1}H-NMR (600 MHz, $CDCl_3$, 27 °C): δ = 11.60 (s, 1H; NHCO), 8.72 (s, 1H; NHCOC(CH_3)$_3$), 8.02 – 7.98 (m, 2H; ArH), 7.92 – 7.89 (m, 2H; ArH), 7.80 – 7.77 (m, 2H; ArH), 7.63 – 7.51 (m, 3H; ArH), 7.47 – 7.43 (m, 2H; ArH), 7.39 – 7.35 (m, 2H; ArH), 7.34 – 7.30 (m, 2H; ArH), 6.93 (dd, $^{3}J_{H3',H4'}$ = 8.3 Hz, $^{3}J_{H3',H2'}$ = 5.0 Hz, 1H; C3′H), 6.76 (s, 1H; C8H), 6.49 (dd, $^{3}J_{H2',H3'}$ = 5.0 Hz, $^{3}J_{H2',H1'}$ = 1.8 Hz, 1H; C2′H), 6.01 (d, $^{3}J_{H1',H2'}$ = 1.8 Hz, 1H; C1′H), 5.89 (dd, $^{3}J_{H2'',H1''}$ = 6.2 Hz, $4J_{H2'',H5''}$ = 1.4 Hz, 1H; C2″H), 5.80 – 5.77 (m, 1H; C1″H), 5.27 (dd, $^{3}J_{H2''',H3'''}$ = 10.0 Hz, $^{3}J_{H2''',H1'''}$ = 7.9 Hz, 1H; C2‴H), 4.93 (d, $^{3}J_{H1''',H2'''}$ = 7.8 Hz, 1H; C1‴H), 4.77 (dt, $^{3}J_{H4',H3'}$ = 8.3 Hz, $^{3}J_{H4',H5'a}$ = $^{3}J_{H4',H5'b}$ = 3.2 Hz, 1H; C4′H), 4.71 (dd, $^{2}J_{H5'a,H5'b}$ = 12.4 Hz, $^{3}J_{H5'a,H4'}$ = 3.2 Hz, 1H; C5′Ha), 4.64 (dd, $^{2}J_{H5'b,H5'a}$ = 12.4 Hz, $^{3}J_{H5'b,H4'}$ = 3.3 Hz, 1H; C5′Hb), 4.59 (dd, $^{3}J_{H5'',H4''}$ = 5.2 Hz, $^{4}J_{H5'',H2''}$ = 1.9 Hz, 1H; C5″H), 4.04 – 4.00 (m, 2H; C4″H, C4‴H), 3.95 – 3.89 (m, 2H; NHC*H*$_2$, C3″H), 3.81 – 3.76 (m, 2H; NHC*H*$_2$, C3‴H), 3.73 (dd, $^{2}J_{H6'''a,H6'''b}$ = 9.9 Hz, $^{3}J_{H6'''a,H5'''}$ = 7.8 Hz, 1H; C6‴Ha), 3.70 (dd, $^{2}J_{H6'''b,H6'''a}$ = 9.9 Hz, $^{3}J_{H6'''b,H5'''}$ = 6.0 Hz, 1H; C6‴Hb), 3.51 – 3.46 (m, 1H; C5‴H), 1.72 (s, 3H; CCl(C*H*$_3$)$_2$), 1.68 (s, 3H; CCl(CH_3)$_2$), 1.35 (s, 9H; COC(C*H*$_3$)$_3$), 0.93 (s, 9H; SiC(CH_3)$_3$), 0.88 (s, 9H; SiC(CH_3)$_3$), 0.88 (s, 9H; SiC(CH_3)$_3$), 0.84 (s, 9H; SiC(CH_3)$_3$), 0.20 (s, 3H; $SiCH_3$), 0.13 (s, 3H; $SiCH_3$),

0.11 (s, 3H; $SiCH_3$), 0.10 (s, 3H; $SiCH_3$), 0.08 (s, 3H; $SiCH_3$), 0.07 (s, 3H; $SiCH_3$), 0.06 (s, 3H; $SiCH_3$), 0.05 (s, 3H; $SiCH_3$) ppm.

^{13}C-NMR (150 MHz, $CDCl_3$, 27 °C): δ = 179.9 ($COC(CH_3)_3$), 170.6 ($COCCl(CH_3)_2$), 166.1(ArCO), 166.0 (ArCO), 165.1 (ArCO), 157.7 (C6), 146.9 (C4), 145.2 (C2), 135.0 (C2″), 133.7 (2C; Ar), 133.4 (Ar), 132.9 (C1″), 129.8 (2C; Ar), 129.7 (2C; Ar), 129.3 (2C; Ar), 129.1 (Ar), 128.9 (Ar), 128.8 (Ar), 128.6 (2C; Ar), 128.5 (2C; Ar), 128.3 (2C; Ar), 120.2 (C8), 120.0 (C7), 105.3 (C5), 100.3 (C1‴), 89.4 (C1′), 80.9 (C4″), 78.2 (C4′), 75.4 (C5‴), 74.8 (C5″), 74.6 (C2′), 74.1 (C2‴), 74.0 (C3‴), 71.5 (C4‴), 70.7 (C3′), 65.9 (C3″), 65.1 ($CCl(CH_3)_2$), 61.8 (C5′), 61.4 (C6‴), 43.2 ($NHCH_2$), 40.2 ($COC(CH_3)_3$), 30.5 ($CCl(CH_3)_2$), 30.4 ($CCl(CH_3)_2$), 26.9 (3C; $COC(CH_3)_3$), 26.2 (6C; $SiC(CH_3)_3$), 26.1 (3C; $SiC(CH_3)_3$), 25.8 (3C; $SiC(CH_3)_3$), 18.6 ($SiC(CH_3)_3$), 18.5 ($SiC(CH_3)_3$), 18.1 (2C; $SiC(CH_3)_3$), −3.6 ($SiCH_3$), −3.8 ($SiCH_3$), −4.2 ($SiCH_3$), −4.5 ($SiCH_3$), −4.8 ($SiCH_3$), −5.1 ($SiCH_3$), −5.2 ($SiCH_3$), −5.3 ($SiCH_3$) ppm.

HRMS (ESI+) *m/z*: calc. for $[C_{77}H_{114}O_{17}N_5ClSi_4 + H]^+$: 1528.7048, found: 1528.7044.

7-Desaza-7-((((3*S*,4*R*,5*S*)-4-*O*-(β-D-galactopyranosyl)-5-hydroxy-cyclopent-1-en-3-yl)-amino)methyl)guanosin (galQ) [2]

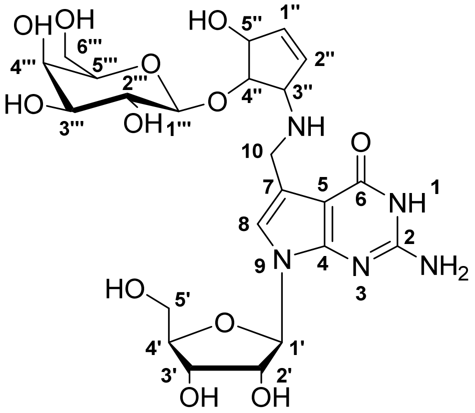

To a solution of 7-Desaza-7-((((3*S*,4*R*,5*S*)-4-*O*-(3,4,6-tri-*O*-*tert*-butyldimethylsilyl-2-*O*-(2-chlorisobutyryl)-β-D-galactopyranosyl)-5-*O*-(*tert*-butyldimethylsilyl)cyclopent-1-en-3-yl)amino)-methyl)-2-pivaloylamino-2′,3′,5′-tri-*O*-benzoylguanosine (85.0 mg, 55.0 µmol, 1.0 eq.) in DCM (2 mL) Triethylamine trihydrofluoride (0.3 mL) was added and the solution was stirred at room temperature for 4 d. Methoxytrimethylsilane (1 mL) was added and the solution was stirred for 1 h before the solvent was removed *in vacuo*. The residue was taken up in a solution of NaOMe in methanol (0.5M, 3 mL) and the resulting solution was stirred for 2 d at room temperature. After neutralization with HCl (2M) the solvent was removed in vacuo. HPLC-purification of the crude product gave 15 mg of a 2.5:1 mixture of the galQ hydroacetate (28%) and triethylammonium acetate.

HPLC-Purification Preparative HPLC column (*250/10 Nucleosil*): Buffer system: buffer A (0.1 M $HNEt_3OAc$ in H_2O), buffer B (0.1 M $HNEt_3OAc$ in H_2O/MeCN 20/80), gradient: 0% buffer B → 8% buffer B, 45 min, flow 5 mL/min;
Analytical HPLC column (*250/4 Nucleodur*): buffer system buffer A (0.1 M $HNEt_3OAc$ in H_2O), buffer B (0.1 M $HNEt_3OAc$ in H_2O/MeCN 20/80), gradient: 0% buffer B → 8% buffer B, 45 min, flow:0.5 mL/min, $\mathbf{R}_t$ = 25.2 min.

^{1}H-NMR (600 MHz, CD_3OD, 27 °C): δ = 7.13 (s, 1H; C8H), 6.17 (ddd, $^3J_{H1'',H2''}$ = 6.3, $^3J_{H1'',H5''}$ = 2.6 Hz, $^4J_{H1'',H3''}$ = 2.2 Hz, 1H; C1″H), 6.11 (dd, $^3J_{H2'',H1''}$ = 6.4 Hz, $^4J_{H2'',H5''}$ = 1.5 Hz, 1H;C2″H), 5.93 (d, $^3J_{H1',H2'}$ = 5.9 Hz, 1H; C1′H), 4.69 (ddd, $^3J_{H5'',H4''}$ = 5.2 Hz, $^3J_{H5'',H1''}$ = 2.8 Hz, $^4J_{H5'',H2''}$ = 1.3 Hz, 1H; C5″H), 4.49 (d, $^3J_{H1''',H2'''}$ = 7.7 Hz, 1H; C1‴H), 4.43 (t, $^3J_{H2',H1'}$ = 5.6 Hz, 1H;C2′H), 4.31 (s, 2H; NHC*H*$_2$), 4.31 – 4.29 (m, 1H; C3″H), 4.23 (dd, $^3J_{H3',H2'}$ = 5.3 Hz, $^3J_{H3',H4'}$ = 3.6 Hz, 1H; C3′H), 4.19 (dd, $^3J_{H4'',H3''}$ = 6.2 Hz, $^3J_{H4'',H5''}$ = 5.4 Hz, 1H; C4″H), 4.03 (q, $^3J_{H4',H3'}$ = $^3J_{H4',H5'a}$ = $^3J_{H4',H5'b}$ = 3.4 Hz, 1H; C4′H), 3.85 (dd, $^3J_{H4''',H3'''}$ = 3.3 Hz, $^3J_{H4''',H5'''}$ = 0.6 Hz, 1H; C4‴H), 3.81 (dd, $^2J_{H5'a,H5'b}$ = 12.2 Hz, $^3J_{H5'a,H4'}$ = 3.1 Hz, 1H; C5′Ha), 3.76 (dd, $^2J_{H6'''a,H6'''b}$ = 11.2 Hz, $^3J_{H6'''a,H5'''}$ = 7.0 Hz, 1H; C6‴Ha), 3.73 (dd, $^2J_{H6'''b,H6'''a}$ = 11.2 Hz, $^3J_{H6'''b,H5'''}$ = 5.1 Hz, 1H; C6‴Hb), 3.72 (dd, $^2J_{H5'b,H5'a}$ = 12.2 Hz, $^3J_{H5'b,H4'}$ = 3.6 Hz, 1H; C5′Hb), 3.64 (dd, $^3J_{H2''',H3'''}$ = 9.7 Hz,

$^{3}J_{H2''',H1'''}$ = 7.6 Hz, 1H; C2‴H), 3.62 (ddd, $^{3}J_{H5''',H6'''a}$ = 6.9 Hz, $^{3}J_{H5''',H6'''b}$ = 5.2 Hz, $^{3}J_{H5''',H4'''}$ = 1.2 Hz, 1H; C5‴H), 3.52 (dd, $^{3}J_{H3''',H2'''}$ = 9.7 Hz, $^{3}J_{H3''',H4'''}$ = 3.3 Hz, 1H; C3‴H), 1.94 (s, 3H, CO_2CH_3) ppm.

^{13}C-NMR (150 MHz, CD_3OD, 27 °C): δ = 162.4 (C6), 154.4 (C2), 153.8 (C4), 136.9 (C1″), 131.5 (C2″), 120.2 (C8), 111.9 (C7), 105.5 (C1‴), 100.6 (C5), 89.7 (C1′), 86.6 (C4′), 84.8 (C4″), 77.0 (C5‴), 75.8 (C2′), 74.8 (C5″), 74.6 (C3‴), 72.4 (C2‴), 72.2 (C3′), 70.3 (C4‴), 65.8 (C3″), 63.3 (C5′), 62.4 (C6‴), 43.7 (NCH_2), 22.3 ($CO_2\mathit{C}H_3$) ppm.

HRMS (ESI−) *m/z*: calc. for $[C_{23}H_{33}O_{12}N_5 + Cl]^-$: 606.1820, found: 606.1820.

1H-NMR of GalQ (2)

a) Full spectrum

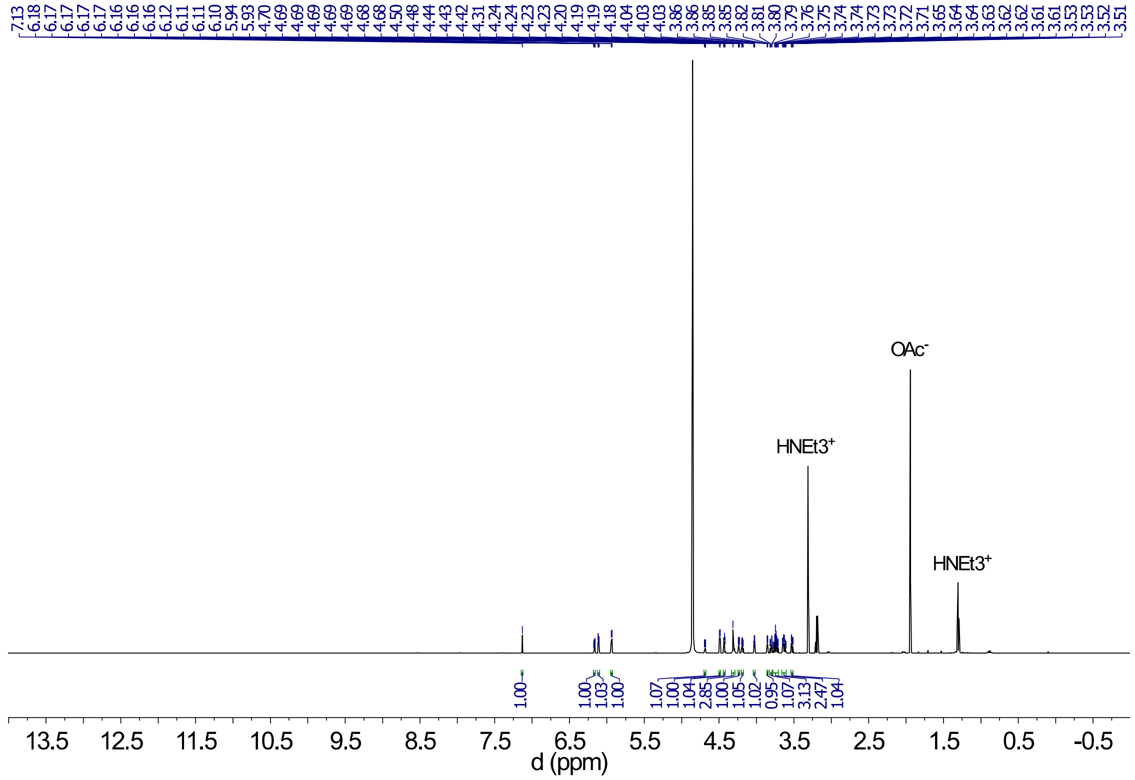

b) Part A zoomed in

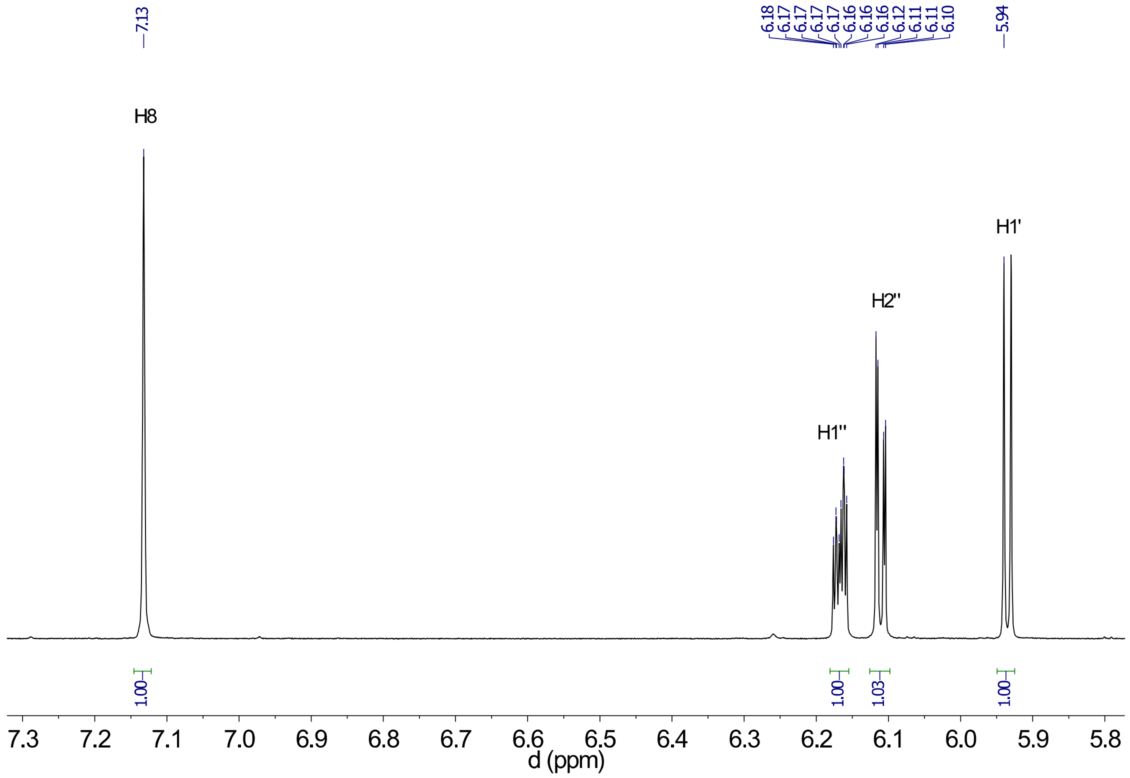

b) Part B zoomed in

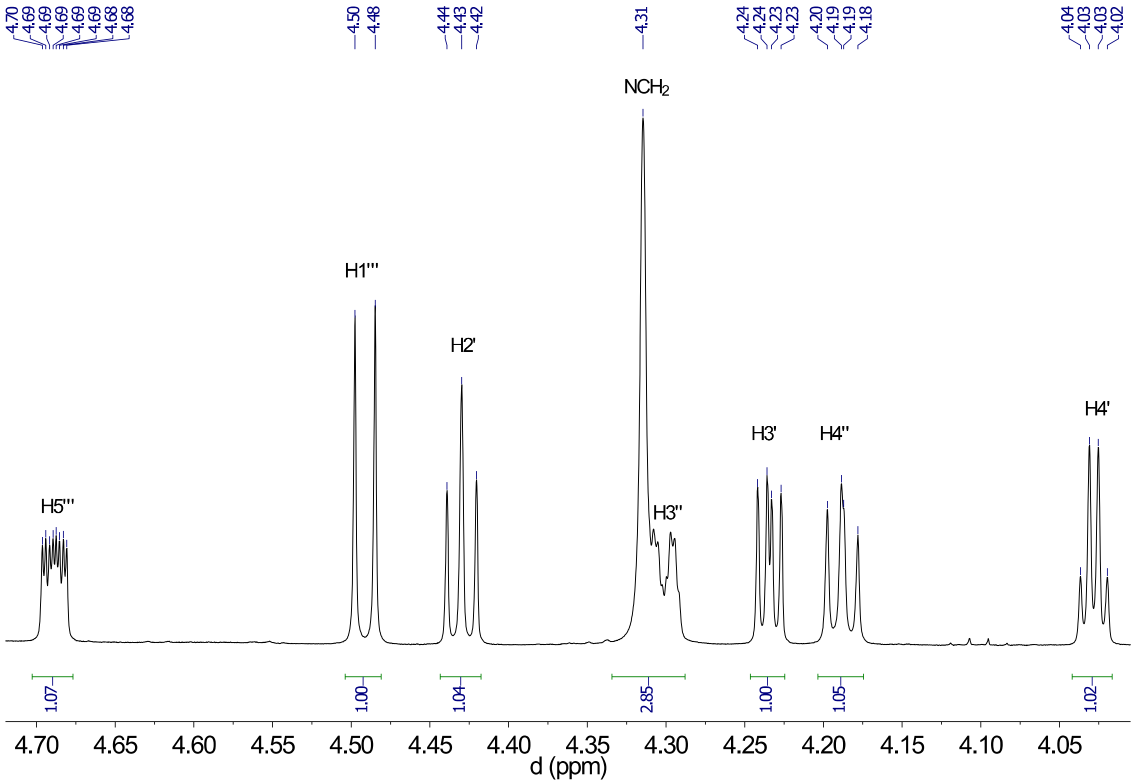

c) Part C zoomed in

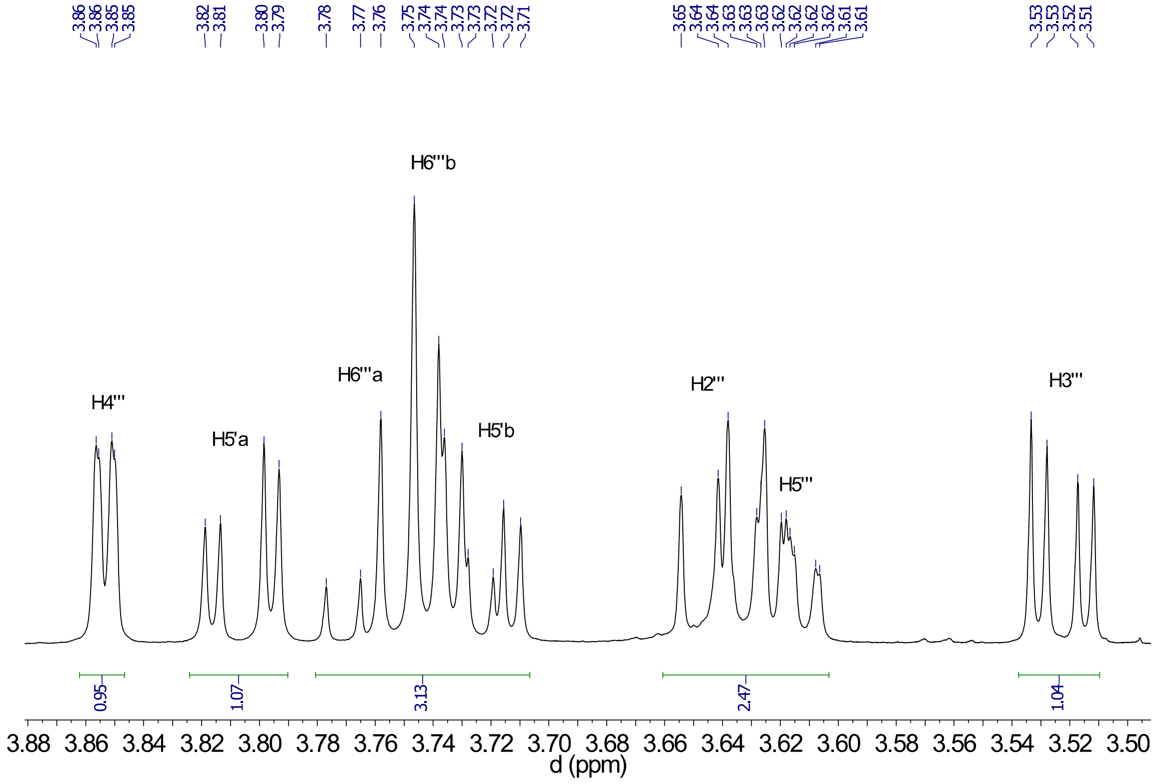

^{13}C-NMR of GalQ (2)

a) Full spectrum

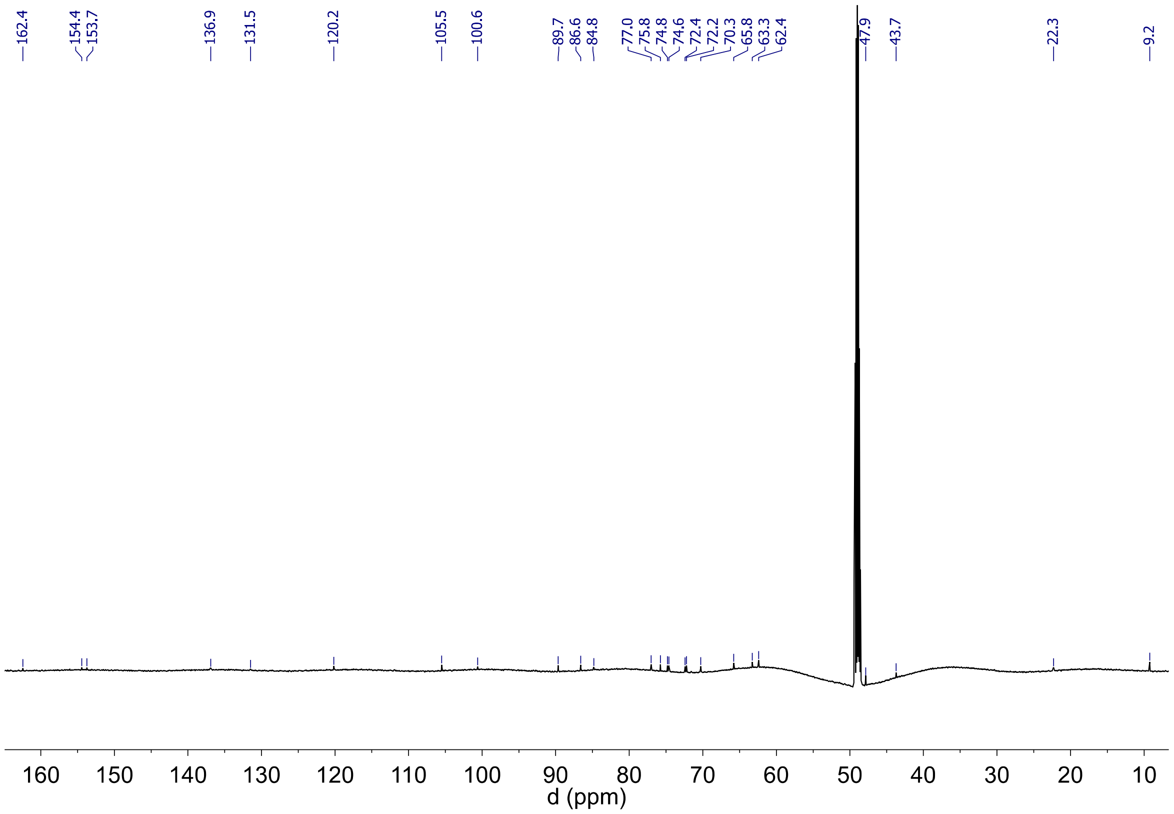

b) Part A zoomed in

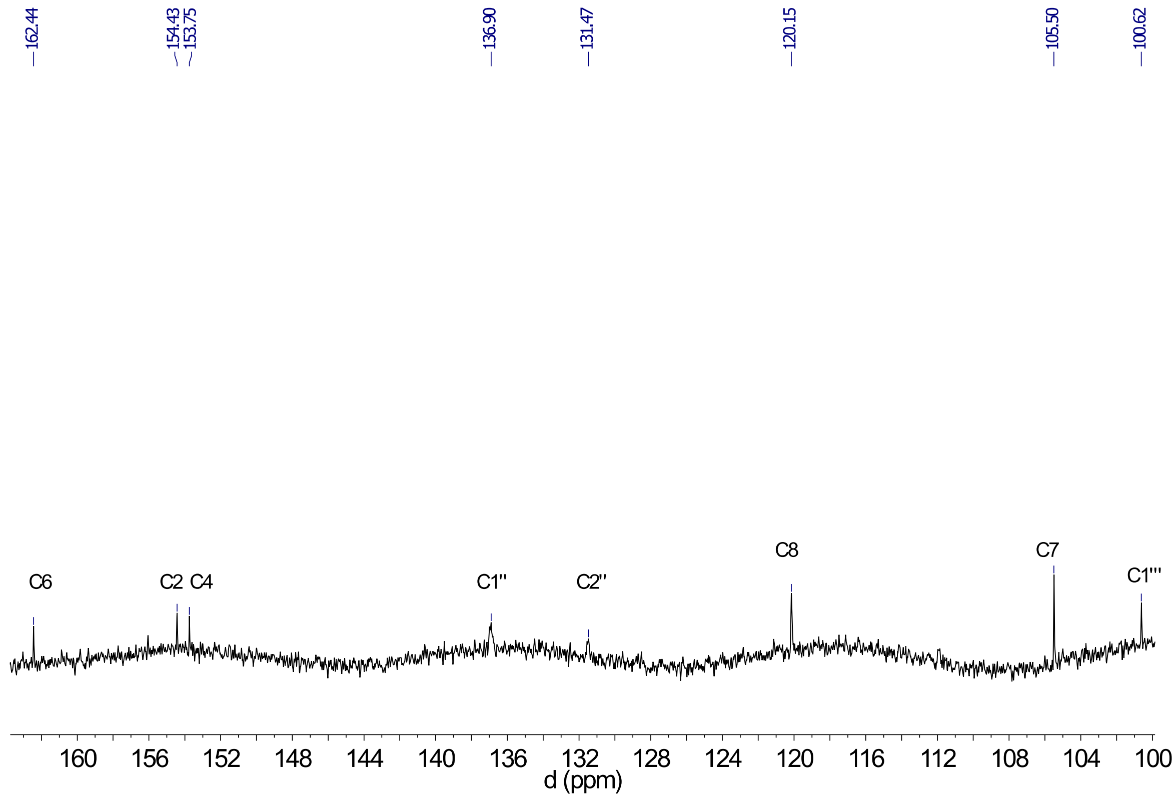

b) Part B zoomed in

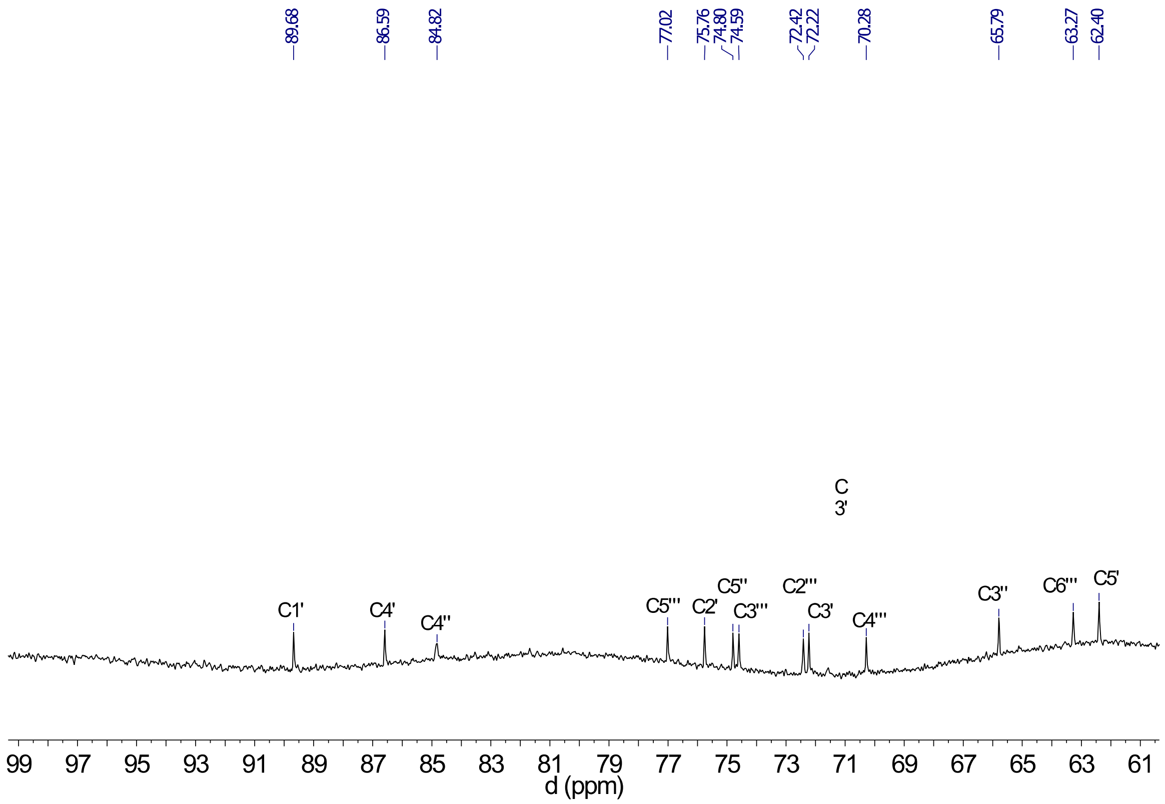

c) Part C zoomed in

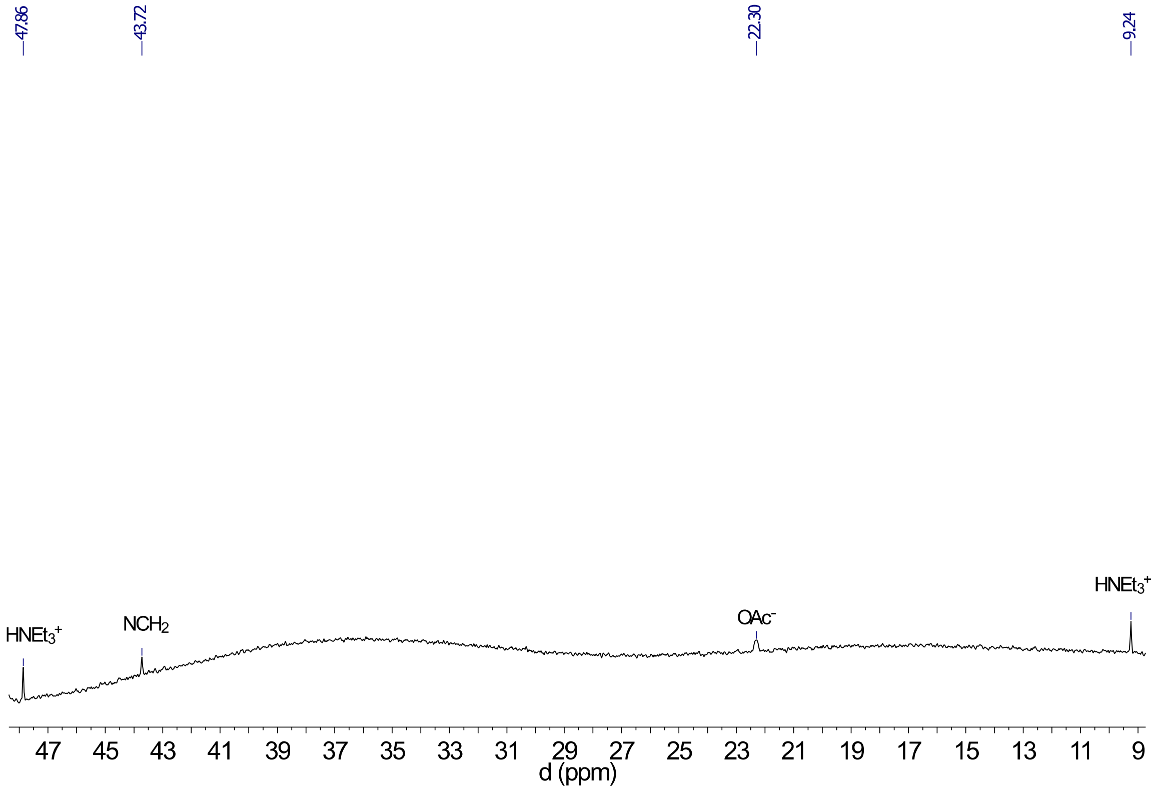

References

[1] Y. H. Chionh, C.-H. Ho, D. a. Pruksakorn, *Nucleic Acids Res.* **2013**, *41*, e168--e168.

[2] M. Heiss, V. F. Reichle, S. Kellner, *RNA Biol.* **2017**, *14*, 1260--1268.

[3] R. Hauenschild, L. Tserovski, K. Schmid, K. Thring, M.-L. Winz, S. Sharma, K.-D. Entian, L. Wacheul, D. L. J. Lafontaine, J. Anderson, J. Alfonzo, A. Hildebrandt, A. Jschke, Y. Motorin, M. Helm, *Nucleic Acids Res.* **2015**, *43*, gkv895.

[4] D. Crich, T. J. Ritchie, *Carbohydr. Res.* **1989**, *190*, C3.

[5] K. C. Nicolaou, S. A. Snyder, D. A. Longbottom, A. Z. Nalbandian, X. Huang, *Chem. - A Eur. J.* **2004**, *10*, 5581--5606.

[6] L. H. Zhang, J. Duan, Y. Xu, W. R. Dolbier, *Tetrahedron Lett.* **1998**, *39*, 9621--9622.

[7] D. Vonlanthen, C. J. Leumann, *Synthesis (Stuttg).* **2003**, 1087--1090.

[8] H. Ovaa, B. Lastdrager, J. D. C. a. Code, *J. Chem. Soc. Perkin 1* **2002**, *2*, 2370--2377.

[9] F. Klepper, E.-M. Jahn, V. Hickmann, T. Carell, *Angew. Chemie Int. Ed.* **2007**, *46*, 2325--2327.
